# Thallium adsorption onto phyllosilicate minerals†

**DOI:** 10.1039/d2em00028h

**Published:** 2022-05-24

**Authors:** Andreas Voegelin, Silvan Wick, Numa Pfenninger, Stefan Mangold, Bart Baeyens, Maria Marques Fernandes

**Affiliations:** Eawag, Swiss Federal Institute of Aquatic Science and Technology Ueberlandstrasse 133 CH-8600 Duebendorf Switzerland andreas.voegelin@eawag.ch; Karlsruhe Institute of Technology Hermann-von-Helmholtz Platz 1 D-76344 Eggenstein-Leopoldshafen Germany; Paul Scherrer Institute Forschungsstrasse 111 CH-5232 Villigen PSI Switzerland

## Abstract

The adsorption of thallium (Tl) onto phyllosilicate minerals plays a critical role in the retention of Tl in soils and sediments and the potential transfer of Tl into plants and groundwater. Especially micaceous minerals are thought to strongly bind monovalent Tl(i), in analogy to their strong binding of Cs. To advance the understanding of Tl(i) adsorption onto phyllosilicate minerals, we studied the adsorption of Tl(i) onto Na- and K-saturated illite and Na-saturated smectite, two muscovites, two vermiculites and a naturally Tl-enriched soil clay mineral fraction. Macroscopic adsorption isotherms were combined with the characterization of the adsorbed Tl by X-ray absorption spectroscopy (XAS). In combination, the results suggest that the adsorption of Tl(i) onto phyllosilicate minerals can be interpreted in terms of three major uptake paths: (i) highest-affinity inner-sphere adsorption of dehydrated Tl^+^ on a very low number of adsorption sites at the wedge of frayed particle edges of illite and around collapsed zones in vermiculite interlayers through complexation between two siloxane cavities, (ii) intermediate-affinity inner-sphere adsorption of partially dehydrated Tl^+^ on the planar surfaces of illite and muscovite through complexation onto siloxane cavities, (iii) low-affinity adsorption of hydrated Tl^+^, especially in the hydrated interlayers of smectite and expanded vermiculite. At the frayed edges of illite particles and in the vermiculite interlayer, Tl uptake can lead to the formation of new wedge sites that enable further adsorption of dehydrated Tl^+^. On the soil clay fraction, a shift in Tl(i) uptake from frayed edge sites (on illite) to planar sites (on illite and muscovite) was observed with increasing Tl(i) loading. The results from this study show that the adsorption of Tl(i) onto phyllosilicate minerals follows the same trends as reported for Cs and Rb and thus suggests that concepts to describe the retention of (radio)cesium by different types of phyllosilicate minerals in soils, sediments and rocks are also applicable to Tl(i).

## Abstract 2

We studied the extent and mode of thallium adsorption onto several phyllosilicate minerals. Thallium, in analogy to cesium, strongly adsorbs onto micaceous phyllosilicates in soils and sediments via complexation in siloxane cavities.

## Graphical abstract

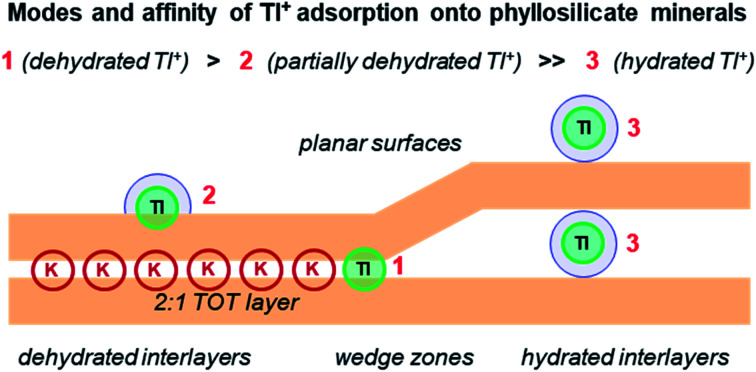

**Environmental significance**

The adsorption of thallium onto phyllosilicate minerals critically impacts on the retention and solubility of this potentially toxic element in soils and sediments. In this work, we studied thallium adsorption onto a range of environmentally relevant phyllosilicate minerals through a combination of macroscopic and synchrotron spectroscopic experiments. The results demonstrate the link between the mode and affinity of thallium uptake and confirm the notion that thallium is strongly retained by micaceous phyllosilicate minerals, in analogy to cesium. Model concepts regarding the retention of radiocesium by phyllosilicate minerals in soils and sediments are thus transferable to thallium and the assessment of its fate and impact in pristine and contaminated environments.

## 1. Introduction

Thallium (Tl) is a potentially toxic trace metal that can pose a serious threat to human and environmental health.^1^ In the environment, Tl dominantly occurs as monovalent Tl(i), more rarely as trivalent Tl(iii) in poorly soluble Tl_2_O_3_ (avicennite) or adsorbed onto hexagonal birnessite.^3–5^ In natural waters over the common pH and *E*_h_ range, the Tl^+^ cation is the prevalent and thermodynamically most stable Tl species.^2,3^ Monovalent Tl(i) exhibits chalcophilic and lithophilic character. Its lithophilic properties are due to the fact that the Tl^+^ cation has a similar ionic radius and a similarly low hydration enthalpy as K^+^, Rb^+^ and Cs^+^ cations. Accordingly, monovalent Tl(i) often shows a reactivity similar to K, Rb and Cs^6–8^ and can substitute K in a wide range of minerals, including mica and micaceous clay minerals.^2,9,10^

From contaminated soils or sediments, Tl may be released into groundwater^11,12^ or be taken up by plants,^13–15^ and may thereby also enter the human food chain.^16,17^ The mobility and bioavailability of Tl in soils and sediments are dependent on its solubility, which is controlled by adsorption processes. Because of the geochemical properties of Tl, illite and related clay minerals have long been assumed to be key adsorbents of Tl in soils and sediments.^18–20^ In a study on a naturally Tl-rich soil developed on mineralized parent rock, we used X-ray absorption spectroscopy (XAS) to characterize the speciation of Tl and found that most Tl in topsoils was associated with illite (or other micaceous minerals).^4^ More recently, we concluded that most geogenic Tl in this soil was retained by fixation in the interlayers of illite, a minor fraction by adsorption as exchangeable Tl.^21^ A laboratory study on the adsorption of Tl onto purified natural illite^8^ showed that the adsorption of Tl in the presence of competing cations can be described by a 3-site cation exchange model that has originally been developed to model the highly selective adsorption of Cs^+^ and Rb^+^ onto illite.^22^ In this model, a limited number of high-affinity sites (“frayed edge sites”, FES) is assumed to represent the adsorption of dehydrated Tl^+^ (or Cs^+^, Rb^+^, K^+^) in the wedge a the frayed edges of illite platelets.^23^ A high number of low-affinity sites on the other hand is assumed to account for the adsorption of Tl^+^ on planar surfaces (planar sites, “PS”). The third type of site required to reproduce macroscopic adsorption isotherms is characterized by intermediate affinity and abundance, but its mechanistic interpretation is uncertain (“type-2 sites”). Cation exchange selectivity coefficients derived for Tl^+^ adsorption onto illite using the 3-site cation exchange model fell between reported selectivity coefficients of Cs^+^ and Rb^+^.^8^ XAS data supported the interpretation that Tl^+^ strongly adsorbed as dehydrated cation at frayed edge sites. Based on these results, and on the known geochemical analogy of Tl with Cs and Rb, it is plausible that other micaceous minerals that are known to strongly bind Cs^+^ and Rb^+^, such as muscovite,^24–26^ weathered biotite,^27^ vermiculite,^28,29^ illite-interlayered smectite,^30^ or glauconite^31^ are also important adsorbents for Tl. To date, however, only limited data exists on the uptake of Tl by muscovite or vermiculite.^20,32,33^

The aim of the present study was to contribute to a better understanding of the adsorption of Tl(i) onto mica and (micaceous) clay minerals. To this end, adsorption isotherms for Tl(i) were measured on Na- and K-exchanged illite, two muscovites and two vermiculites as well as on smectite. Adsorbed Tl was analyzed by Tl L_III_-edge XAS to monitor variations in the mode of Tl uptake between minerals and as a function of Tl loading. Furthermore, we also studied the adsorption of Tl onto a purified clay mineral assemblage from a naturally Tl-rich topsoil.

## 2. Materials and methods

### 2.1. Phyllosilicate minerals used for Tl adsorption experiments

Illite du Puy (IdP) was purified and prepared as homoionic Na-exchanged IdP (Na–IdP; 19.8 g L^−1^; 100 mM NaCl) or K-exchanged IdP (K–IdP; 28.0 g L^−1^; 10 mM KCl) suspensions as described previously.^8^ Wyoming smectite (SWy-2) was purchased from the Source Clays Repository of the Clay Minerals Society (Chantilly, VA, USA) and was prepared as homoionic Na-exchanged SWy-2 suspension (Na–SWy; 20.1 g L^−1^; 100 mM NaCl) as described in previous work.^34^ The Na–IdP, K–IdP and Na–SWy suspensions were stored at 4 °C in the dark for further use.

Adsorption experiments with muscovite and vermiculite were performed two times with different samples and slightly different laboratory protocols (A and B). Natural muscovite A (Musc-A; unknown origin) and thermally exfoliated vermiculite A (Verm-A; Vermex M, 2–4 mm particle size; Isola Vermiculite AG, Switzerland) were provided by Kurt Barmettler from the Soil Chemistry Group at ETH Zurich (Switzerland). Natural muscovite B (Musc-B; Aspanger Bergbau und Mineralwerke GmbH & Co KG, Austria) and thermally exfoliated vermiculite B (Verm-B; Thermax®, Austria) were kindly provided by Michael Plötze from ETH Zurich (Switzerland). The vermiculite Verm-B has previously been used to study Cs adsorption^35^ and the effect of sample pre-treatment on its cation exchange capacity (CEC).^36^

For the adsorption experiments, about 1 g of Musc-A, Verm-A and Verm-B were ground in a ball mill using a tungsten carbide jar (Retsch MM200; 17 hertz; 30 seconds). Musc-B was used as received. The minerals were repeatedly Na-exchanged in 1 M NaNO_3_, washed several times with doubly deionized (DDI) water, and freeze dried for further use.

A soil clay mineral fraction (SCF) was extracted from a naturally Tl-rich soil and treated to remove organic matter and oxides as described previously.^21^ The Na-exchanged SCF was stored as suspension in the dark at 4 °C for further use.

### 2.2. Mineral characterization

The illite used in this work has been used in an earlier Tl adsorption study,^8^ and has also been used to study the protonation and metal uptake of illite.^37,38^ Details on the purification procedure and the composition of Na–IdP can be found in these earlier studies. Another batch of illite from the same locality (Le Puy-en-Velay, France) has previously been used to study the adsorption of Cs.^39^ The muscovite and vermiculite samples were analyzed by X-ray diffraction (XRD) and scanning electron microscopy coupled with energy dispersive X-ray detection (SEM-EDX). The combined results confirmed that the samples consisted of muscovite or vermiculite (ESI Sections 1.1 and 1.2†). The soil clay mineral fraction (SCF) was extracted from a topsoil sample (PI 00–20) examined in an earlier study on the speciation of geogenic Tl in soils from Erzmatt (Switzerland).^4^ This soil sample had a pH of 6.5 (10 mM CaCl_2_) and contained 54% clay, 2.6% organic carbon, and 120 mg kg^−1^ geogenic Tl. The clay mineral fraction of this soil sample consists primarily of illite/muscovite, hydroxy-interlayered vermiculite/chlorite and interstratified illite-smectite.^4^ XRD data collected on the oriented Mg- and K-exchanged SCF and SEM-EDX data collected on the SCF qualitatively confirmed this analysis (ESI Sections 1.1 and 1.2†). Semiquantitative laboratory micro-XRF analysis of the SCF indicated a geogenic Tl content of 260 mg kg^−1^. The cation exchange capacities (CEC) of all samples are listed in Table 1 (see ESI Section 1.3† for details on source and method of determination).

**Table 1:** Cation exchange capacity (CEC) of the phyllosilicate minerals and linear regression equations describing the log-scale adsorption isotherms (eqn (1); logarithmic form of the Freundlich equation; values in parentheses indicate uncertainty). Log-scale distribution coefficients (*K*_d_/(L kg^−1^)) and cation exchange selectivity coefficients for Tl–Na exchange (*K*_c,Tl–Na_) were calculated for a dissolved Tl concentration of 3.2 μM (log(*C*/(mol L^−1^)) = −5.5), using the linear regression to calculate the corresponding adsorbed Tl concentrations. See ESI Section 1.3 for determination methods or source of the CEC values

Mineral	CEC (mmol kg^−1^)	Linear regression equations			Log(*K*_d_/(L kg^−1^))	Log(*K*_c,Tl–Na_)
		Log(*K*_F_)	*n*_F_	*r* ^2^		
Na–IdP	260	0.52 (0.20)	0.48 (0.04)	0.98	3.4	3.0
K–IdP	260	1.49 (0.08)	0.85 (0.02)	1.00	2.3	—
Na–SWy	860	0.91 (0.13)	0.79 (0.03)	1.00	2.0	1.1
Musc-A	121	0.88 (0.12)	0.61 (0.02)	1.00	3.0	3.0
Musc-B	144	1.10 (0.20)	0.61 (0.04)	1.00	3.3	3.1
Verm-A	1320	5.45 (0.45)	1.53 (0.09)	0.99	2.6	1.4
Verm-B	1590	13.5 (3.1)	3.09 (0.63)	0.89	2.0	0.8
SCF	290	0.71 (0.08)	0.57 (0.02)	1.00	3.1	2.6

### 2.3. Adsorption experiments

#### Adsorption experiments with Na–IdP, K–IdP and Na–SWy-2

Experiments with Na–IdP and Na–SWy-2 were performed in 0.1 M NaCl background electrolyte, the experiments with K–IdP in 0.01 M KCl background electrolyte. 10 mL of the clay suspensions (∼200 mg clay mineral) were added to 25 mL of background electrolyte in 40 mL polypropylene (PP) tubes (Beckman Coulter) (resulting in ∼5 g L^−1^ clay mineral in suspension). Subsequently, Tl(i) was spiked using a 2.5 mM TlNO_3_ stock solution to achieve nominal (maximum) loadings of 300, 1000, 3000, 10 000 and 30 000 mg kg^−1^ Tl(i) (in case of 100% Tl adsorption). Subsequently, the concentration of the background electrolyte was readjusted to the desired value by addition of a 10-fold concentrated electrolyte solution. After shaking by hand, the pH was measured and, if lower than 6.4, adjusted to 7.0 using NaOH. Subsequently, the suspensions were placed on an overhead shaker and reacted for about 14 days at room temperature. After phase separation by ultra-centrifugation (108 000*g* (max), 30 000 rpm, 1 h, Beckman Coulter Avanti J30i High-Performance Centrifuge), the pH was recorded in all supernatants before two 7 mL aliquots were sampled and acidified with 100 μL 65% HNO_3_ (Suprapur) for analysis by inductively coupled plasma mass spectrometry (ICP-MS).

#### Adsorption experiments with muscovite and vermiculite

For the experiments with Musc-A and Verm-A, 50 mL polyethylene (PE) tubes were filled with 35 mL of 0.1 M NaNO_3_ background electrolyte. Subsequently, 200 mg of the freeze-dried samples Musc-A or Verm-A were added to the tubes. In the case of Verm-A, initial pH values of pH ∼ 9.8–9.9 were lowered to pH 6.8–7.4 by adding 50–60 μL 1 M HCl. Tl(i) was spiked using a 2.5 mM TlNO_3_ solution to achieve nominal Tl(i) loadings of 300 to 30 000 mg kg^−1^ Tl. The suspensions were reacted for 7 days at room temperature on an overhead shaker. After centrifugation (2700*g*, 4000 rpm, 5 min, Rotofix 32 Hettich), 20 mL aliquots of the supernatant were filtered, acidified and diluted for ICP-MS analysis, and pH was recorded in the residual solution.

For adsorption experiments with Musc-B and Verm-B, the 50 mL PE tubes were filled with 26 mL of 0.1 M NaNO_3_, followed by the addition of 150 mg of the dried solids. If the pH of the suspensions was >8, it was set to ∼7 by addition of 1 M HCl. Subsequently, Tl(i) was spiked using a 10 mM TlNO_3_ stock solution to achieve nominal Tl(i) loadings of 300 to 30 000 mg kg^−1^. After reaction for 7 days on an overhead shaker, the suspensions were centrifuged (400 rpm, 5 min), a 10 mL aliquot was filtered (0.2 μm nylon membrane) and acidified for analysis by ICP-MS, and pH was recorded in the remaining supernatant. The adsorption experiments with Musc-B were conducted in duplicates. After adsorption, the second sample for each loading was extracted with 0.1 M Ca(NO_3_)_2_. For this purpose, the supernatant after adsorption was decanted, and 50 mL of 0.1 M Ca(NO_3_)_2_ were added to the PE tubes. After reaction for 2 h on a table shaker and subsequent centrifugation, aliquots of the supernatant were filtered and acidified for analysis by ICP-MS, and the solids collected for XAS analysis.

#### Adsorption experiment with soil clay fraction

To 35 mL of 0.1 M NaNO_3_ background electrolyte in 50 mL PE tubes, 4.7 mL of the SCF stock suspension were added, corresponding to about 250 mg of SCF. Subsequently, Tl(i) was spiked using a 10 mM TlNO_3_ stock solution (in 0.1 M NaNO_3_) to achieve nominal Tl(i) loadings of about 200 to 20 000 mg kg^−1^ Tl. The suspensions were reacted for 7 days. After centrifugation, a filtered aliquot (0.2 μm nylon membrane) of the supernatant was sampled for ICP-MS analysis, and the final pH was recorded in the remaining supernatant.

### 2.4. X-ray absorption spectroscopy

#### Preparation of Tl-loaded mineral samples for XAS

After the adsorption experiments, the Tl-loaded moist solids were gently dewatered with a paper tissue and prepared as pastes in acrylic glass holders closed with polyimide tape on the front and the back. The mounted pastes were stored in the dark at 4 °C in closed containers together with a moist tissue until analysis at the synchrotron. Note that names of XAS samples and spectra in this manuscript consist of the phyllosilicate mineral name followed by a number indicating the Tl loading in mg kg^−1^.

#### XAS measurements

The Tl-loaded phyllosilicates and reference minerals were analyzed by XAS at the Tl L_III_-edge at several XAS beamlines. At all beamlines, a Si(111) monochromator was used for X-ray energy selection, ionization chambers to measure the incident and transmitted X-ray photon intensity, and a solid-state fluorescence detector to record the Tl Lα fluorescence line. Samples with Tl loadings up to 10 000 mg kg^−1^ Tl were in general recorded in fluorescence mode, samples with Tl loadings of 10 000 and higher in transmission mode. The X-ray photon energy was aligned by measuring the X-ray near-edge structure (XANES) spectrum of Tl_2_O_3_ (maximum of whiteline at 12 688 eV) and/or elemental Se(0) (K-edge; first maximum of first derivative at 12 658 eV). All samples were measured at room temperature, few selected samples also at 20 K. An overview over all XAS measurements is given in Table S4.†

#### Tl-loaded minerals

The Tl-loaded Na–IdP samples were analyzed at the SuperXAS beamline at the Swiss Light Source (SLS, Paul Scherer Institute (PSI), Villigen, Switzerland), the Tl-loaded Musc-A, Verm-A and K–IdP samples at the XAS beamline at the Synchrotron Radiation Source at the Karlsruhe Institute of Technology (KIT, Eggenstein-Leopoldshafen, Germany), the Tl-loaded SCF at the Dutch Belgian Beamline (DUBBLE) at the European Synchrotron Radiation Facility (ESRF, Grenoble, France), and Tl-loaded Musc-B and Verm-B samples at the SAMBA beamline at the French national synchrotron light source Soleil (Gif-sur-Yvette, France). The sample Na–IdP 3200 was also recorded at 20 K at the SAMBA beamline.

#### Reference spectra

Natural Tl-containing Ba–muscovite (“oellacherite”) with ∼200 mg kg^−1^ Tl from the Binn valley in Switzerland was kindly provided by Beda Hofmann (Natural History Museum Berne, Switzerland).^40,41^ The reference spectra of the Tl-containing Ba–muscovite and of aqueous Tl^+^ (10 mM TlNO_3_) were recorded at the SuperXAS beamline. For the Ba-rich muscovite, an individual platelet was analyzed at a 35° angle between the electronic field vector of the incident beam and the platelet surface to minimize polarization effects.^42^ The same muscovite platelet in the same orientation was also analyzed at 20 K at the SAMBA beamline.

#### XAS data extraction and analysis

The software code Athena was used for raw data extraction and for the analysis of XANES spectra by linear combination fitting (LCF), the software code Artemis for the analysis of extended X-ray absorption fine structure (EXAFS) spectra by shell fitting.^43^

## 3. Results and discussion

### 3.1. Adsorption data

**Fig. 1:**
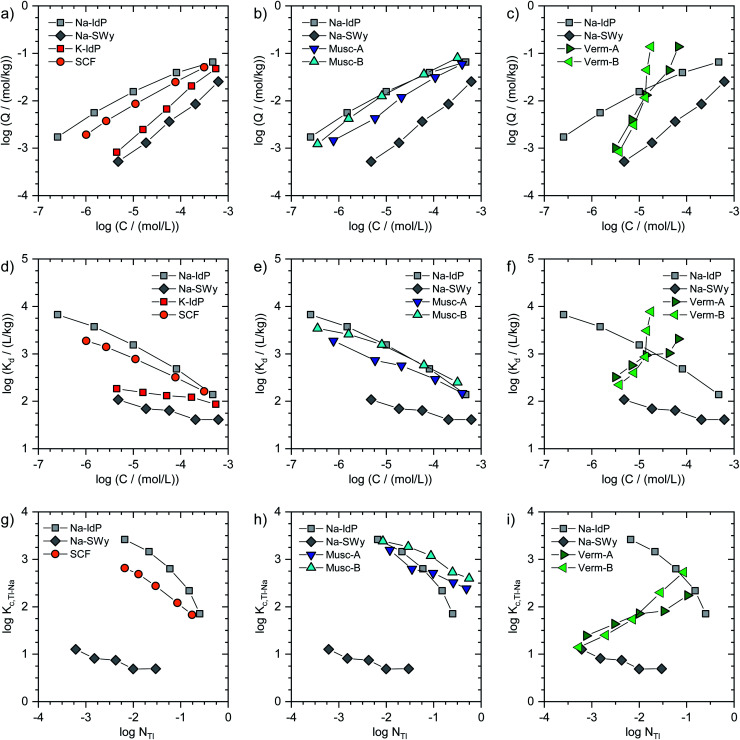
(a–c) Log-scale Tl adsorption isotherms for Na–IdP, K–IdP, Na–SWy, Musc-A, Musc-B, Verm-A, Verm-B and SCF shown as adsorbed amount (*Q*) *versus* dissolved concentration (*C*). (d–f) Log-scale Tl adsorption isotherms shown as distribution coefficient (*K*_d_) *versus* dissolved concentration. (g–i) Log-scale conditional cation exchange selectivity coefficients for Tl–Na exchange (*K*_c,Tl–Na_) *versus* equivalent fraction of adsorbed Tl (*N*_Tl_). The adsorption data for Na–IdP and Na–SWy are shown in all panels for comparison. Adsorption data were measured in 100 mM Na^+^ electrolyte for all sorbents except for K–IdP, where 10 mM K^+^ electrolyte was used; therefore, no data can be shown for K–IdP in panel (g).

In Fig. 1, the Tl adsorption data for all phyllosilicate minerals are shown on the log-scale in three formats: (i) adsorbed amounts (*Q*) *versus* dissolved concentrations (*C*) (Fig. 1a–c), (ii) distribution coefficients (*K*_d_) *versus* dissolved concentrations (*C*) (Fig. 1d–f), and (iii) cation exchange selectivity coefficients (*K*_c,Tl–Na_) *versus* equivalent fractions of adsorbed Tl (*N*_Tl_) (Fig. 1g–i). The tabulated results are given in the ESI (Table S3).† The adsorption data for Na–IdP and Na–SWy are shown in all panels for comparison with the other adsorbents. Final pH values in the suspensions ranged between pH 6.5 and pH 7.1 for Na–IdP, K–IdP, Na–SWy (one sample pH 7.9), Musc-A and SCF, and between pH 7.8 and pH 8.6 for Musc-B, Verm-A and Verm-B (Table S3†).

The log-scale adsorption isotherms (Fig. 1a–c) were described by linear regression equations that correspond to the logarithmic form of the Freundlich isotherm equation:

1 Log(*Q*/(mol kg^−1^)) = log *K*_F_ + *n*_F_ × log(*C*/(mol L^−1^))

where *Q* represents the adsorbed amount of Tl in mol kg^−1^, *C* the dissolved concentration in mol L^−1^, *K*_F_ the Freundlich constant, and *n*_F_ the Freundlich coefficient. The respective regression parameters are listed in Table 1. The same regression parameters can also be used to describe the distribution coefficients *K*_d_ (=*Q*/*C*) as a function of the dissolved Tl concentrations (Fig. 1d–f):

2 Log(*K*_d_/(L kg^−1^)) = log *K*_F_ + (*n*_F_ − 1) × log(*C*/(mol L^−1^))

For brevity, we in the following refer to log(*Q*/(mol kg^−1^)) as log *Q*, to log(*C*/(mol L^−1^)) as log *C*, and to log(*K*_d_/(L kg^−1^)) as log *K*_d_. Interpolated log *K*_d_ values for a Tl concentration of 3.2 μM (log *C* = −5.5) are listed in Table 1.

#### Illites Na–IdP and K–IdP

**Fig. 2:**
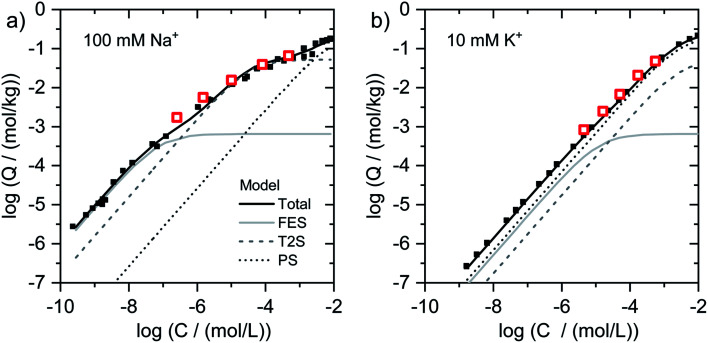
Isotherms for Tl adsorption onto (a) Na–IdP and (b) K–IdP as a function of dissolved Tl. Data from the present work (open red squares) are shown together with published adsorption data over larger concentrations ranges from Wick *et al.* (2018)^8^ (black squares) and corresponding model calculations based on the 3-site cation exchange model for illite (FES = frayed edge sites, T2S = type-2 sites; PS = planar sites).

For illite, the adsorption data obtained for Na–IdP in 100 mM Na^+^ and for K–IdP in 10 mM K^+^ background electrolyte (Fig. 1a and d) closely matched to adsorption data from our earlier study in which radioactive ^204^Tl was used to quantify Tl adsorption onto the same illite over much larger concentration ranges (Fig. 2).^8^ In this former study, cation exchange selectivity coefficients for Tl adsorption onto illite have been parameterized in the framework of the 3-site cation exchange model previously developed to model Cs uptake by illite. In the model calculations shown in Fig. 2, the adsorption of Tl onto Na–IdP within the concentration range covered in the current study is mainly attributed to type-2 sites (T2S, Fig. 2), and the adsorption of Tl onto K–IdP to planar sites (PS). The model calculations thus are in line with the mechanistic interpretation that the more than one order of magnitude higher Tl loading of Na–IdP in 100 mM Na^+^ than of K–IdP in 10 mM K^+^ electrolyte is due to effective competition of K^+^ for high-affinity cation exchange sites.^8^

For the other adsorbents studied in this work, the available adsorption data did not allow to derive parameters for detailed (multisite) adsorption models. Nevertheless, conditional cation exchange selectivity coefficients that treat the entire CEC of the adsorbents as a single type of cation exchange site could be derived and interpreted in terms of their dependence on the equivalent fraction of adsorbed Tl. The exchange of adsorbed Na by Tl can be described by the cation exchange reaction

3 NaX + Tl^+^ ↔ TlX + Na^+^

where Tl^+^ and Na^+^ represent the dissolved metal cations and TlX and NaX the cations adsorbed to exchange sites X^−^. Based on this reaction equation, conditional cation exchange selectivity coefficients for Tl–Na exchange (*K*_c,Tl–Na_) were calculated as:

4 *K*_c,Tl–Na_ = (*N*_Tl_ × *C*_Na_)/(*N*_Na_ × *C*_Tl_)

*C*_Tl_ and *C*_Na_ correspond to the dissolved concentrations of Tl^+^ and Na^+^, and *N*_Tl_ and *N*_Na_ to the equivalent fractions of adsorbed Na and Tl. *N*_Tl_ was calculated from the adsorbed amount of Tl and the CEC (*N*_Tl_ = *Q*_Tl_/CEC), and *N*_Na_ was calculated assuming that exchange sites were covered by either Tl or Na (*i.e.*, *N*_Na_ = 1 − *N*_Tl_). In Table 1, log(*K*_c,Tl–Na_) values are listed for a Tl concentration of 3.2 μM (log *C* = −5.5), using eqn (1) to derive the respective log *Q*. In Fig. 1g–i, the adsorption data for all adsorbents (except K–IdP where adsorption was measured in 10 mM K^+^ electrolyte) are shown as log(*K*_c,Tl–Na_) *versus* log(*N*_Tl_) (in the following referred to as log *K*_c,Tl–Na_ and log *N*_Tl_, respectively). The data for Na–IdP and Na–SWy are shown in all panels for comparison. In the case of Na–IdP (Fig. 1g), log *K*_c_,_Tl–Na_ decreased by about 1.6 log units from lowest to highest log *N*_Tl_ (Fig. 1g). This marked decrease reflects the stepwise saturation of an increasing number of adsorption sites with decreasing cation exchange selectivity, as represented by the progressive saturation of FES, T2S and PS in the 3-site cation exchange model calculations (Fig. 2).

#### Smectite Na–SWy

The Tl adsorption isotherm for Na–SWy indicated a much lower Tl adsorption affinity at low concentrations than for Na–IdP (Fig. 1a and d), as also observed in an earlier study on the adsorption of Tl onto Na- and Ca-saturated illite and Wyoming smectite.^44^ The maximum loading of Tl on Na–SWy reached only about 3% of its CEC (Fig. 1g). With increasing log *N*_Tl_, the log *K*_c,Tl–Na_ decreased by about 0.5 log units, with an average log *K*_c,Tl–Na_ of 0.85 ± 0.17 (Fig. 1g). The relatively low log *K*_c,Tl–Na_ at even the lowest log *N*_Tl_ suggests that high affinity adsorption sites were not relevant over the probed concentration range. Indeed, a study on Cs adsorption onto another smectite indicated that high affinity adsorption sites similar to FES only contributed to Cs adsorption at Cs loadings far below the lowest Tl loadings in the present work.^30^ For Cs–Na exchange on the dominant cation exchange sites that represent most of the CEC, on the other hand, a log *K*_c,Cs–Na_ of 1.7 ± 0.7 had been derived,^30^ about 0.8 log units larger than the log *K*_c,Tl–Na_ for Na–SWy reported above. For Cs–Na and Rb–Na exchange on Wyoming bentonite (similar to Na–SWy used in the present work), log *K*_c,Cs–Na_ and log *K*_c,Rb–Na_ values of ∼1.2 and ∼0.5 have been reported for *N*_Cs_ or *N*_Rb_ of ∼0.05,^45,46^ compared to a log *K*_c,Tl–Na_ of 0.7 observed in the current study for *N*_Tl_ = 0.03 (Fig. 1g). Another study derived a log *K*_c,Cs–Na_ of 0.8 for Na-saturated Wyoming smectite at a low Na background electrolyte concentration of 5 mM that allowed for a larger contribution of low-affinity Cs adsorption.^47^ Overall, the comparison of reported cation exchange selectivity coefficients for Cs–Na and Rb–Na exchange on Na-saturated smectite with the log *K*_c,Tl–Na_ derived in the current work indicated that the adsorption affinity of Tl on smectite falls between the affinities of Cs and Rb, as previously reported for cation exchange on illite.^8^

#### Muscovites Musc-A and Musc-B

The isotherms for Tl adsorption onto the muscovites Musc-A and Musc-B are shown in Fig. 1b and e. The adsorption isotherms of the two muscovites differed by less than 0.5 log units in terms of Tl loading at a given dissolved Tl concentration. At low dissolved Tl concentrations, the Tl loadings were slightly lower than for Na–IdP, but markedly higher than for Na–SWy, showing that the muscovites can bind Tl with very high affinity; as reflected by log *K*_d_ values of 3.0 (Musc-A) and 3.3 (Musc-B) for *C* = 3.2 μM (Table 1). At the highest dissolved Tl concentrations, the muscovites adsorbed similar amounts of Tl as Na–IdP (Fig. 1b and e). In combination with their lower CEC values, this suggested that the muscovites can strongly bind Tl even at high equivalent fractions (Fig. 1h). The extraction of one set of Tl-loaded Musc-B samples with 0.1 M Ca(NO_3_)_2_ (at a high solution-to-solid ratio of 333 mL g^−1^) released only between 1.4% of the adsorbed Tl at the lowest loading and 19% at the highest loading, confirming that Tl was strongly adsorbed.

#### Vermiculites Verm-A and Verm-B

The adsorption isotherms for the vermiculites Verm-A and Verm-B showed a markedly different trend than the other adsorption isotherms: with increasing (residual) dissolved Tl concentration, the adsorbed amounts of Tl increased more than proportionally. This corresponds to an increase in sorption affinity with loading (Fig. 1c and f) that is also reflected in the increase of the log *K*_c,Tl–Na_ (Fig. 1i). At the lowest Tl loadings, the adsorption of Tl onto the Na-exchanged vermiculites resembled the adsorption of Tl onto Na–SWy. Towards the highest Tl loadings, the adsorption affinity of Tl for the vermiculites even exceeded the one for Na–IdP (Fig. 1f and i). Considering earlier results on the binding of Cs and Tl by vermiculite,^28,33,48,49^ this trend was attributed to Tl-induced interlayer collapse and the generation of an increasing number of high-affinity sorption sites in fresh wedge zones.

#### Soil clay mineral fraction SCF

The data for Tl adsorption onto the soil clay mineral fraction closely resembled the adsorption data of Na–IdP and the two muscovites in terms of Tl loading, sorption affinity, and cation exchange selectivity (Fig. 1a, d and g), in line with the identification of illite and muscovite as major components of the soil clay mineral fraction.

### 3.2. X-ray absorption spectroscopy

In this section, the Tl L_III_-edge XANES and EXAFS reference spectra of Tl-containing Ba–muscovite and aqueous Tl^+^ are first discussed. Subsequently, an overview over spectral trends in the sample spectra is given, followed by a step-wise comparison and interpretation of spectral trends among the different minerals and as a function of Tl loading, and in relation to the macroscopic adsorption data. An overview over all analyzed spectra is given in Table S4.†

#### Reference spectra

**Fig. 3:**
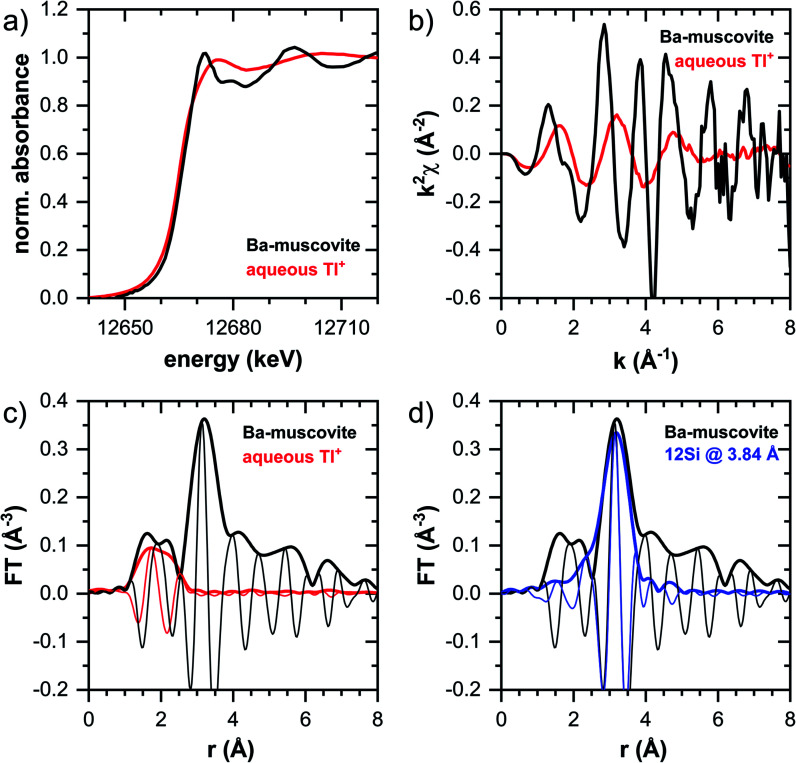
XANES and EXAFS reference spectra of Tl-rich Ba–muscovite and of 10 mM aqueous Tl^+^: (a) XANES spectra, (b) *k*^2^-weighted EXAFS spectra, (c) Fourier-transform of *k*^2^-weighted EXAFS spectra (magnitudes: thick lines; imaginary parts: thin lines). (d) Fourier-transform of EXAFS spectrum of Tl-rich Ba–muscovite compared with shell-fit involving 12 Si atoms at a distance of 3.84 Å from Tl.

The Tl L_III_-edge reference spectra of Tl(i)-containing Ba–muscovite and of aqueous Tl^+^ are shown in Fig. 3. The absorption edges of both XANES spectra are characteristic of monovalent Tl(i) (as also evidenced by comparison with the spectrum of trivalent Tl(iii) in Tl_2_O_3_; Fig. S5†). The marked differences in the XANES and EXAFS spectra of the two Tl(i) reference spectra reflect the difference in local Tl coordination geometry between hydrated aqueous Tl^+^ and dehydrated Tl^+^ in the interlayer of Ba–muscovite.

The EXAFS spectrum of Tl(i) in Ba–muscovite features much higher amplitudes than the spectrum of hydrated aqueous Tl^+^ (Fig. 3). In the Fourier-transform, the magnitude of the first peak is very low for both compounds. This can be explained by substantial disorder in first-shell Tl–O coordination due to the lone electron pair of the Tl^+^ cation^50^ and the destructive interferences arising from a wide spread in Tl–O interatomic distances. The EXAFS spectrum of aqueous Tl^+^ has previously been described by 2 short and 2 long Tl–O paths with an average Tl–O distance of 2.875 Å.^50^ The shift of the imaginary part of the first-shell of Tl in Ba–muscovite to higher *r*-values points to a larger average first-shell Tl–O distance than in aqueous Tl^+^. This difference could be expected based on the K–O distance of 12-fold coordinated K^+^ in illite of 3.109 Å^51^ and the larger ionic radius of 12-fold coordinated Tl^+^ (1.70 Å) than K^+^ (1.64 Å).^52^

The analysis of the EXAFS of Tl-containing Ba–muscovite shown in Fig. 3 and of second spectrum recorded at 20 K by shell fitting indicated that the second FT peak can be modelled with 12 Si atoms at an average distance of 3.84 Å from the absorbing Tl atom, based on a Tl–Si single-scattering path derived from the structure of illite (ESI Section 3, Table S6 and Fig. S17†). The fitted distance was in good agreement with an average second-shell K–Si distance of 3.774 Å in illite^51^ and an EXAFS-derived Cs–Si distance of Cs incorporated into illite of 3.99 Å,^53^ considering that the ionic radius of 12-fold coordinated Tl^+^ (1.70 Å) falls in between the ionic radii of K^+^ (1.64 Å) and Cs^+^ (1.88 Å).^52^ We thus interpreted the peak around ∼3.2 Å in the Fourier-transformed EXAFS spectrum of Ba-rich muscovite as arising from 12-fold Si-coordinated Tl(i) between two adjacent siloxane cavities in the interlayer.

#### Sample spectra

**Fig. 4:**
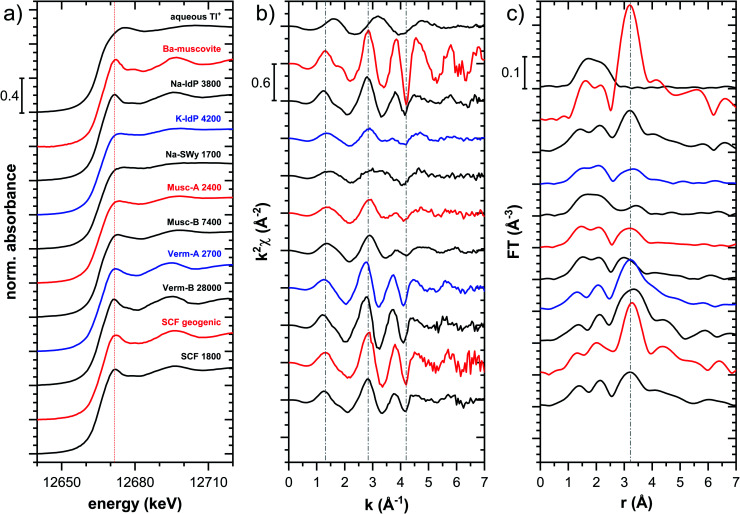
Tl L_III_-edge XANES (a), EXAFS (b) and Fourier-transformed EXAFS (c) spectra of selected Tl-loaded illite (Na–IdP; K–IdP), smectite (Na–SWy), muscovite (Musc-A; Musc-B), and vermiculite (Verm-A; Verm-B) samples and of a soil clay fraction (SCF) with geogenic Tl and with additional sorbed Tl. Numbers in spectra labels indicate the Tl loading of the respective phyllosilicate in mg kg^−1^. Vertical lines serve to guide the eye.

**Fig. 5:**
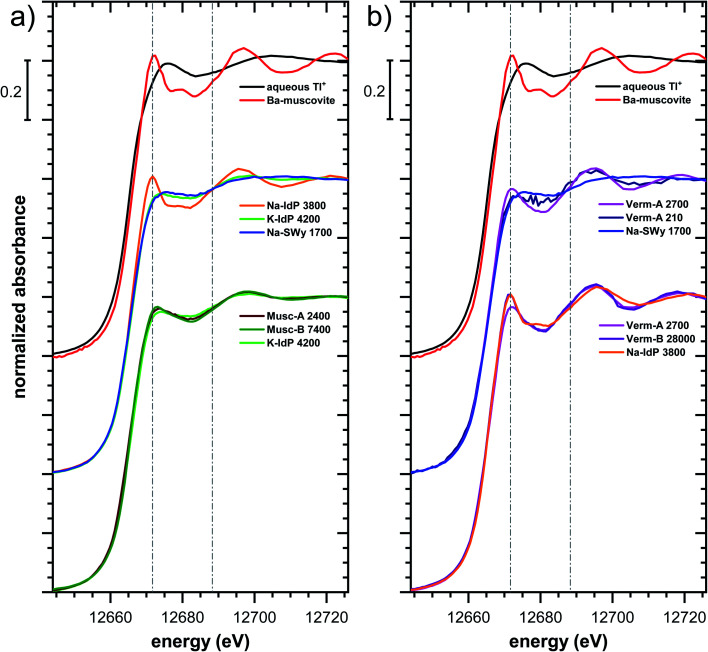
Comparison of the Tl L_III_-edge XANES spectra of selected Tl-loaded phyllosilicate samples. Numbers in spectra labels indicate the Tl loading of the respective phyllosilicate in mg kg^−1^. Vertical lines serve to guide the eye. An enlarged version of figure focusing on post-edge oscillations is provided in Fig. S16.†

**Fig. 6:**
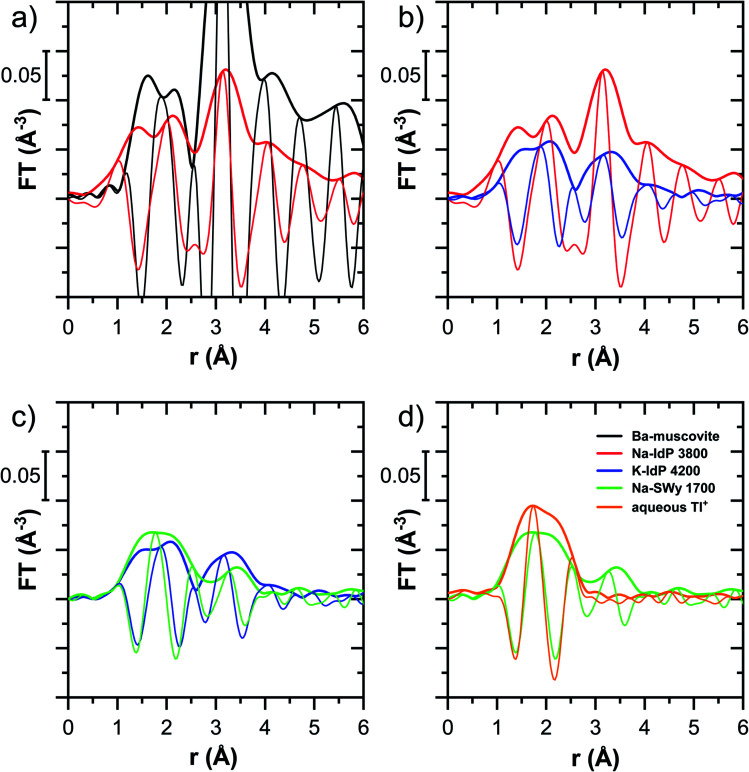
Fourier-transformed *k*^2^-weighted Tl L_III_-edge EXAFS spectra of Tl-containing Ba–muscovite, Tl-loaded Na–IdP and K–IdP, Na–SWy and of aqueous Tl^+^; sequentially pair-wise compared (a–d; thick lines indicate magnitude, thin lines imaginary part). Numbers in sample names indicate the Tl loading of the phyllosilicates in mg kg^−1^.

**Fig. 7:**
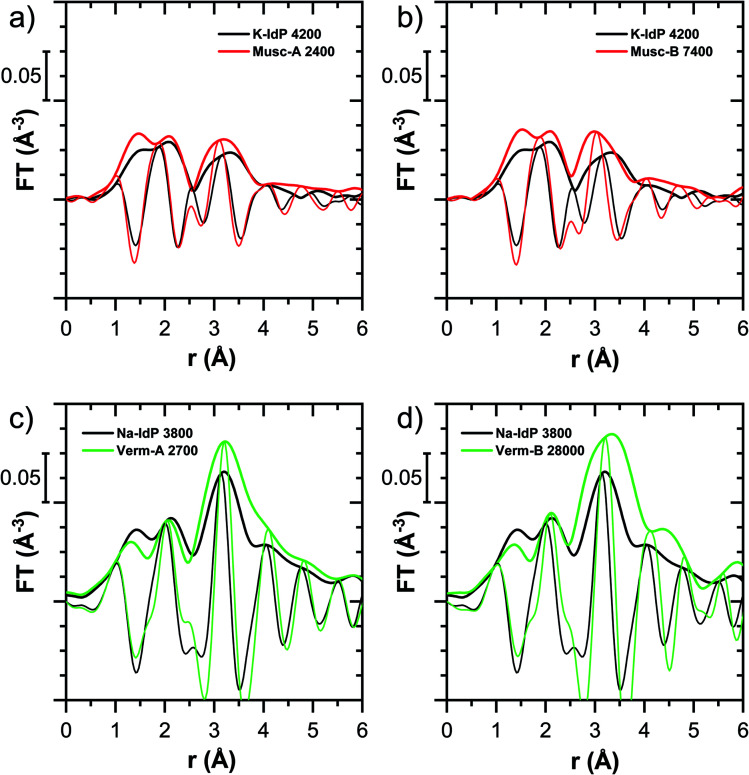
Fourier-transformed *k*^2^-weighted Tl L_III_-edge EXAFS spectra of (a and b) Tl-loaded muscovites Musc-A and Musc-B compared to Tl-loaded K–IdP and of (c and d) Tl-loaded vermiculites Verm-A and Verm-B compared to Tl-loaded Na–IdP. Numbers in sample names indicate the Tl loading of the phyllosilicates in mg kg^−1^.

Tl L_III_-edge XANES spectra were recorded for all Tl-loaded phyllosilicate samples (Fig. S6–S15†), EXAFS spectra were recorded for a limited subset of samples (Fig. S18–S20†). The XANES and EXAFS spectra of selected samples are shown in Fig. 4. The observed spectral variations are indicative of variations in the mode of Tl(i) binding by the different minerals (illite, smectite, muscovite, vermiculite), as a function of cation saturation (Na–IdP *vs.* K–IdP), and as a function of Tl loading (vermiculites, SCF). In the following paragraphs, these differences are interpreted by stepwise comparisons of selected XANES (Fig. 5 and S16†) and EXAFS (Fig. 6 and 7) spectra.

The XANES and EXAFS spectra of the Tl-loaded phyllosilicate samples (Fig. 4–7, S6–S15 and S18–S20†) lacked detectable spectral contributions of Tl(iii). Considering that the XANES and EXAFS spectra of Tl(iii)-compounds are very distinct (Fig. S5†), this supported the expectation that oxidative uptake of Tl (as on vacancy-containing hexagonal birnessite^5,54^) was not relevant for the studied phyllosilicate minerals.

#### Illite Na–IdP

The XANES spectra of Tl adsorbed onto Na–IdP showed only minor variations as Tl loading was varied from 350 to 13 000 mg kg^−1^ (Fig. S6†), and matched closely to spectra of low amounts of Tl adsorbed onto Na–IdP from our earlier study^8^ (apart from beamline-related differences in X-ray energy resolution). In this earlier study, spectral changes at Tl loadings above ∼3800 mg kg^−1^ pointed to the increasing binding of hydrated Tl^+^ onto Na–IdP. Such a shift was not observed in the present study for two reasons: first, in our earlier study, the background Na concentration significantly decreased at Tl loadings above ∼3800 mg kg^−1^ due to dilution of the background electrolyte by the added Tl stock (which contained no Na), allowing for increasing low-affinity adsorption of hydrated Tl^+^ on planar surfaces. In contrast, in the present study, the background electrolyte concentration was kept constant at 100 mM Na at all Tl loadings, which limited the low-affinity adsorption of hydrated Tl^+^. Second, the highest Tl loading in the present study was about 4 times lower than in the earlier work, also limiting the extent of low-affinity uptake of hydrated Tl^+^.

The EXAFS spectrum Na–IdP 3800 shown in Fig. 4 and 6 was recorded on a sample from our earlier study.^8^ This EXAFS spectrum rather than the one recorded on the analogous sample Na–IdP 3200 from the current study was used for further evaluation because of higher spectral quality. Comparison of the FT EXAFS spectrum of Na–IdP 3800 to the spectrum of Ba–muscovite (Fig. 6a) showed that the peak position and imaginary part of the second FT peak match closely. We therefore attribute the peak at ∼3.2 Å in the Na–IdP 3800 spectrum to contributions of second-shell Si around dehydrated Tl^+^ adsorbed at the frayed edges of illite platelets. Also above and below the second-shell Tl–Si peak, the oscillations in the imaginary part of the FT EXAFS of Na–IdP 3800 matched the oscillations of the spectrum of Tl-containing Ba–muscovite, supporting the interpretation that Tl in Na–IdP is similarly coordinated as in Ba–muscovite. In the case of dehydrated Tl^+^ adsorbed between two siloxane cavities, a Tl–Si coordination number of 12 as for Tl in the interlayer of Ba–muscovite would be expected. The much lower second-shell peak amplitude of Na–IdP 3800 may indicate that the disorder in Tl–Si coordination of Tl adsorbed at frayed edges of illite is much higher than in Ba–muscovite. A shell-fit analysis of the EXAFS spectra of Tl adsorbed onto Na–IdP recorded at room temperature and 20 K showed that the second-shell FT peak could be reasonably reproduced with 12 Si atoms at a distance of 3.95 Å, somewhat longer than in Ba–muscovite, and with higher Debye–Waller factors (ESI Section 3, Fig. S19 and Table S6†). Because this shell-fit was poorly constrained and based on a fixed coordination number, however, we cannot exclude that spectral contributions of Tl with lower degree of Tl–Si coordination limit the second-shell Tl–Si peak amplitude. Such contributions could arise from partially hydrated Tl^+^ that is adsorbed onto single siloxane cavities (with an ideal Tl–Si coordination number of 6) on planar surfaces or in the hydrated interlayer at frayed particle edges. However, we note that spectra of Na–IdP loaded with 300 mg kg^−1^ (1.5 mmol kg^−1^) and 50 mg kg^−1^ (0.25 mmol kg^−1^) Tl did not markedly deviate from the spectrum Na–IdP 3800 (19 mmol kg^−1^ Tl) (Fig. S18†), which suggests that local Tl coordination did not change over a wide range of Tl loadings. Taking further into account that the lowest loading of 0.25 mmol kg^−1^ was below the concentration of highest-affinity cation exchange sites in the 3-site cation exchange model for illite (0.65 mmol kg^−1^),^8^ it is therefore probable that the spectrum Na–IdP 3800 is representative for the coordination of Tl adsorbed at frayed edge sites between two siloxane cavities.

#### Illite K–IdP

The XANES spectra of Tl adsorbed onto K–IdP showed no variation with Tl loading (Fig. S7†), indicating that the mode of Tl uptake did not vary greatly. Compared to Tl adsorbed onto Na–IdP, the spectral features were substantially attenuated (Fig. 5 and 6). In the FT EXAFS spectrum of Tl adsorbed onto K–IdP (K–IdP 4200), the second-shell signal was markedly lower than in the spectrum of Tl adsorbed onto Na–IdP (Na–IdP 3800), but with closely matching peak position and imaginary part (Fig. 6b). Compared to the spectrum Na–IdP 3800, the spectrum K–IdP 4200 revealed a shift of the first-shell Tl–O distance towards lower *r*-value. In combination, these spectral differences indicated a lower degree of Tl–Si coordination and higher degree of hydration of Tl^+^ adsorbed onto K–IdP than Na–IdP. This difference was attributed to the collapse of frayed edges in K–IdP upon K saturation, the resulting loss of high affinity frayed edge sites, and consequently predominant Tl adsorption *via* inner-sphere complexation onto siloxane cavities on planar surfaces (a viable adsorption mode for Cs^+^ on illite based on molecular simulations^55^). This interpretation was also supported by the close similarity of the spectrum K–IdP 4200 to the spectra of Tl adsorbed onto the muscovites Musc-A and Musc-B, as discussed further below.

#### Smectite Na–SWy

The XANES spectra of Tl adsorbed ono Na–SWy did not markedly vary with Tl loading (Fig. S8†), apart from a higher noise level in the lowest loaded sample. The XANES spectrum Na–SWy 1700 exhibited even less pronounced spectral features than the spectrum K–IdP 4200 and resembled the spectrum of aqueous Tl^+^ (Fig. 5). In the FT EXAFS spectrum of Na–SWy 1700, the first-shell peak amplitude and imaginary part closely matched those of aqueous Tl^+^ (Fig. 6d). The second-shell peak was again lower than in the spectrum of the sample K–IdP 4200 (Fig. 6c). The peak was also slightly shifted relative to the peaks of Na–IdP 3800 and K–IdP 4200, possibly due to contributions of backscatterers other than Si that become relevant when the Tl–Si signal amplitude is low. In combination, the XANES and EXAFS spectra of the sample Na–SWy 1700 in combination with the low adsorption affinity of Tl for Na–SWy (Fig. 1a, d and g) suggested that a substantial fraction of the Tl adsorbed onto Na–SWy was hydrated Tl^+^ bound as an outer-sphere complex. On the other hand, the small second-shell signal indicated that part of the Tl was inner-spherically bound in the hydrated interlayers of smectite or on its planar surfaces. These interpretations are in agreement with molecular simulations that have shown that uptake of Cs^+^ in the interlayers of smectite is primarily due to outer-sphere adsorption.^56^

#### Muscovites Musc-A and Musc-B

The XANES spectra of the muscovites did not vary greatly with Tl loading (Fig. S9 and S10†). Both their XANES and EXAFS spectra were similar to the spectrum of Tl^+^ adsorbed onto K–IdP (Fig. 5 and 7a and b). The extraction of 19% of the total adsorbed Tl from the highest-loaded Musc-B sample did not cause detectable changes in the XANES or EXAFS spectra (Fig. S11†). Taken together, this observations suggested that the major share of Tl was strongly adsorbed *via* the same adsorption mechanism, irrespective of Tl loading, and that only a minor fraction of the Tl was adsorbed as weakly-bound hydrated Tl^+^, as also reflected in the high adsorption affinity of Tl on the muscovites at all loadings (Fig. 1c, f and i). We therefore interpreted the macroscopic and spectroscopic results as indicative for predominant inner-sphere adsorption of partially hydrated Tl^+^ onto siloxane cavities on the planar surfaces of the muscovites. For Cs^+^ (and Rb^+^), this mode of adsorption has been documented through synchrotron X-ray reflectivity and molecular simulation studies.^57–62^ The similarity of the EXAFS spectra of Tl^+^ adsorbed onto the muscovites and onto K–IdP in turn supported our interpretation that Tl adsorbed onto K–IdP primarily *via* inner-sphere complexation on its planar surfaces. The shell-fit analysis of the EXAFS spectra Musc-A 2400 and Musc-B 7400 and K–IdP 4200 showed that reasonable fits of the second-shell peak could be achieved with Tl–Si coordination numbers fixed to 6, the value expected for Tl coordinated onto a siloxane cavity (Table S6 and Fig. S20†).

#### Vermiculites Verm-A and Verm-B

For both vermiculites, a change in the XANES spectra was observed with increasing loading that pointed to a change in the mode of Tl uptake (Fig. S12 and S13†). The spectra of the lowest-loaded samples of each vermiculite still showed spectral features reminiscent of the spectrum of Tl adsorbed onto Na–SWy, the higher loaded samples exhibited the spectral features of dehydrated Tl^+^ adsorbed onto Na–IdP, albeit with some differences in the spectral oscillations (Fig. 5, S12 and S13†). LCF analysis showed that the Tl-loaded vermiculite spectra could be described as linear combinations of the spectrum Na–SWy 1700 and the spectrum of the vermiculite sample with highest loading (Table S5, Fig. S12 and S13†). We interpreted the spectrum Na–SWy 1700 as proxy for hydrated Tl^+^ adsorbed in the hydrated vermiculite interlayer, and the spectra of the highest loaded vermiculite samples as proxies for dehydrated Tl^+^ adsorbed at newly formed wedge sites in collapsed parts of the vermiculite interlayer. Based on this interpretation, about half of the total Tl in the lowest-loaded vermiculite samples was adsorbed as hydrated Tl^+^ (57% in Verm-A 210, 48% in Verm-B). The fraction of adsorbed hydrated Tl^+^ became negligible as the Tl loading increased to ∼9000 mg kg^−1^ (3% in Verm-A 8900, 2% in Verm-B 9100). This trend was in close agreement with the adsorption data, which indicated that Tl was only weakly adsorbed at the lowest Tl loadings, similar to Tl adsorption onto Na–SWy, and that Tl adsorption affinity markedly increased with increasing Tl loading (Fig. 1f and i). The comparison of the XANES spectra of the Tl-loaded samples Verm-A 2700, Ver-B 28000 and Na–IdP 3800 revealed consistent differences in the post-edge region between the vermiculites and Na–IdP (Fig. 5). The corresponding FT EXAFS spectra showed that the vermiculite samples featured a slightly more intense and slightly shifted second-shell peak compared to Tl adsorbed onto Na–IdP (Fig. 7c and d). These spectral differences pointed to variations in the local coordination of dehydrated Tl^+^ in the interlayers of vermiculite and illite. However, these differences could not be further resolved by shell-fitting due to the limited data space (ESI Section 3.5, Table S6 and Fig. S20†).

#### Soil clay mineral fraction SCF

**Fig. 8:**
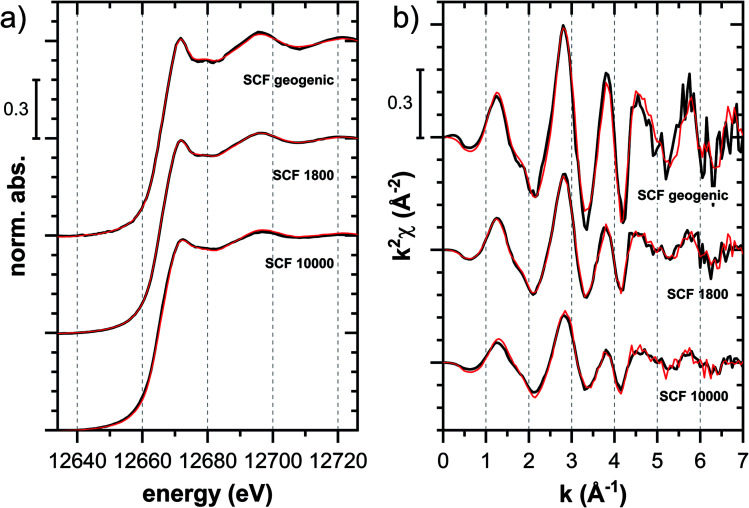
Tl L_III_-edge (a) XANES and (b) EXAFS spectra of a soil clay fraction (SCF) with ∼260 mg kg^−1^ geogenic Tl (SCF geogenic) and with an additional 1800 mg kg^−1^ (SCF 1800) or 10 000 mg kg^−1^ freshly adsorbed Tl (SCF 10000) (black lines) and linear fit reconstructions (red lines). LCF results are listed in Table 2.

In the case of the soil clay mineral fraction SCF, the XANES data revealed gradual spectral changes from geogenic Tl in the unspiked SCF over the SCF samples with lowest to highest Tl loading (Fig. 3, S14 and S15†). The XANES and EXAFS spectra of the unspiked SCF with ∼260 mg kg^−1^ geogenic Tl and of the SCF with 1800 and 10 000 mg kg^−1^ additionally adsorbed Tl are shown in Fig. 8. Both the XANES and EXAFS of the spectrum of geogenic Tl exhibited weaker spectral features than the spectrum of Tl-containing Ba-muscovite (Fig. 3), but more pronounced signal amplitudes than the spectrum of Tl adsorbed at frayed edge sites of illite (Na–IdP 3800). Using LCF, both the XANES and EXAFS signal could be described by a major contribution from the spectrum Na–IdP 3800 and a minor contribution of the spectrum of Ba–muscovite (Fig. 8; Table 2). The XANES and EXAFS spectra of the sample SCF 1800 (with about 85% freshly adsorbed and 15% geogenic Tl) could be reasonably reproduced by a combination of a major contribution of the spectrum Na–IdP 3800 and a minor contribution of the spectrum Musc-A 2400 (Fig. 8). This result in combination with the adsorption data (Fig. 1) showed that the SCF has the capacity to bind freshly added Tl with high-affinity at frayed edge sites. The minor fraction of muscovite in the LCF, on the other hand, suggested that the capacity for high-affinity Tl uptake started to get saturated at a Tl loading of 1800 mg kg^−1^. This became apparent in the spectrum SCF 10000 with lowest signal amplitudes, for which LCF returned a similar or even larger fraction for the Musc-A 2400 than the Na–IdP 3800 reference spectrum. That the sum of fractions in the EXAFS LCF of the sample SCF 10000 was considerably lower than unity (Table 2) could indicate that the Musc-A 2400 reference spectrum did not adequately reproduce the spectral contribution of the least specifically adsorbed Tl. Importantly, reference spectra of Tl adsorbed onto vermiculite (Verm-A 2700, Verm-B 28000) were not required to reproduce the spectra of geogenic and adsorbed SCF.

**Table 2:** Linear combination fit results for the XANES and EXAFS spectra of the samples SCF geogenic, SCF 1800 and SCF 10000 using the reference spectra Ba–muscovite (Ba-musc), Na–IdP 3800 and Musc-A 2400. Fits of the XANES spectra were conducted over the energy range 12 646–12 726 eV, fits of the EXAFS spectra over the *k*-range 3–7 Å^−1^. Fitted fractions were constrained to values between 0 and 1. The LCF reconstructions are shown in Fig. 8. NSSR = normalized sum of squared residuals

	Ba–musc	Na–IdP 3800	Musc-A 2400	Sum	NSSR
**XANES**					
SCF geogenic	0.26	0.74	—	1.00	0.00023
SCF 1800	—	0.73	0.27	1.00	0.00004
SCF 10000	—	0.35	0.66	1.01	0.00050

**EXAFS**					
SCF geogenic	0.33	0.83	—	1.16	0.19
SCF 1800	—	0.78	0.13	0.91	0.12
SCF 10000	—	0.39	0.31	0.69	0.22

### 3.3. Relation between Tl adsorption affinity and uptake mechanisms

Decades of research offer evidence for several distinct modes of Cs adsorption onto clay minerals:^57^ (i) complexation of dehydrated Cs^+^ between two siloxane cavities at the wedge of the frayed edges of illite or weathered mica and at transitions between collapsed and expanded interlayer zones in vermiculite, (ii) complexation of partially hydrated Cs^+^ on a single siloxane cavity on the planar surfaces of muscovite or illite or in the expanded interlayer at frayed edges of illite, (iii) outer- or inner-sphere complexation of Cs^+^ in the hydrated interlayers of swelling clay minerals, and (iv) fixation of Cs^+^ in the interlayer of illite or muscovite over longer times. The combined macroscopic and spectroscopic results of this study show that the adsorption of Tl onto clay minerals occurs in analogy to Cs uptake and reveal a close link between the affinity and mode of Tl adsorption.

For Na-exchanged illite, muscovite and smectite, the macroscopic Tl adsorption data at low dissolved Tl concentrations indicated a decrease in Tl adsorption affinity in the order Na–IdP ∼(≥) Musc-A ∼ Musc-B ≫ Na–SWy (Fig. 1; Table 1). In the XANES spectra, this trend was accompanied by a shift from spectra more closely resembling the spectrum of Tl in the interlayer of Ba–muscovite to spectra more closely resembling the spectrum of aqueous Tl^+^ (Fig. 4 and 5). In analogy, changes in *k*-space EXAFS amplitudes (Fig. 4) reflected a shift of the first-shell Tl–O peak from the distance of Ba–muscovite to the distance of aqueous Tl^+^ and a concomitant decrease of the second-shell Tl–Si peak amplitude (Fig. 4, 6 and 7). These spectral trends are in agreement with predominant adsorption of Tl onto Na–IdP *via* complexation of dehydrated Tl^+^ between two siloxane cavities at frayed edge sites, onto the muscovites *via* the binding of partly hydrated Tl^+^ onto single siloxane cavities on planar surfaces, and onto Na–SWy by mostly outer-sphere binding of hydrated Tl^+^ in hydrated interlayers. The combined results thus point to a decrease in adsorption affinity that is linked to the mode of Tl adsorption.

An analogous relation between adsorption affinity and adsorption mode was also observed for the two vermiculites: low levels of Tl were weakly adsorbed as hydrated Tl^+^ in the hydrated vermiculite interlayers, whereas high levels of Tl led to partial interlayer collapse and strong adsorption of dehydrated Tl^+^ at newly created wedge sites around collapsed interlayer zones (Fig. 1h, 5 and 7).

### 3.4. Cation exchange model sites and their relation to adsorption mechanisms

To describe the adsorption of Cs^+^, Rb^+^ and Tl^+^ onto illite in competition with NH_4_^+^, K^+^, Na^+^ and Ca^2+^, a 3-site cation exchange model has previously been successfully parameterized^8,22,23,39^ The three sites of this model account for: (i) a very small fraction of high-affinity adsorption sites, (ii) an intermediate fraction of medium-affinity adsorption sites, and (iii) a major fraction of adsorption sites with lowest affinity. The same model has also been used to describe Cs adsorption onto biotite, glauconite or micaceous sediments.^24,31,63^ In general, the low-abundance high-affinity sites are assumed to correspond to adsorption sites at the wedge of frayed particle edges (frayed edge sites, “FES”), the high-abundance low-affinity sites to cation exchange sites on planar surfaces (planar sites, “PS”). Since two cation exchange sites were found to be insufficient to adequately describe Cs^+^ and Rb^+^ adsorption isotherms over large concentration ranges, a third type of site with medium-affinity and medium-abundance has been invoked to improve data fits, referred to as “type-2 sites” (“T2S”).^22,23,39^ In the following, we discuss our spectroscopic and macroscopic results with respect to potential relations between adsorption model sites and the mechanisms of Tl uptake.

In the case of Tl adsorbed onto homoionic Na–IdP, we interpreted the XAS of the sample Na–IdP 3800 with 19 mmol kg^−1^ adsorbed Tl as indicative for Tl complexation between two siloxane cavities at frayed particle edges. This interpretation was supported by the observation that no spectral changes were observed in samples with lower Tl loadings of 1.5 and 0.25 mmol kg^−1^ (Fig. S18†), the latter falling below the concentration of FES in the 3-site cation exchange model for illite of 0.65 mmol kg^−1^.^8^ The Tl loading of 19 mmol kg^−1^, however, was much higher than the concentration of FES on illite (derived from adsorption experiments with Na- or Ca-exchanged illite). This suggests that Tl adsorption at particle edges leads to the generation of fresh FES-like wedge sites and to increasing collapse of the frayed edge zone (similar to Tl^+^ induced collapse of vermiculite interlayers). Considering that the interlayers of Na-saturated illite platelets may become hydrated over several nanometers away from the particle edges,^29,53^ the amounts of Tl adsorbed *via* this process can be expected to largely exceed the FES concentration of the 3-site model. In the model calculations, Tl adsorption onto the sample Na–IdP 3800 (19 mmol kg^−1^) was mainly allocated to T2S (site concentration 52 mmol kg^−1^), adsorption onto the sample Na–IdP 50 (0.25 mmol kg^−1^ Tl) mainly to FES (site concentration 0.65 mmol kg^−1^) (Fig. 2). For Na–IdP, the T2S of the 3-site model may therefore account for intermediate-affinity Tl adsorption onto wedge sites that are freshly formed as a consequence of increasing Tl loading and frayed edge collapse.

In the case of K–IdP, the saturation of the illite with K prior to Tl adsorption induced the collapse of the expanded interlayer at particle edges. Accordingly, the adsorption of Tl onto K–IdP in 10 mM K^+^ electrolyte solution was mostly limited to planar surfaces. The XAS data suggested that Tl was predominantly adsorbed by complexation onto siloxane cavities, in analogy to Tl adsorption onto the muscovites. In the 3-site model, the Tl binding onto K–IdP was nearly exclusively described by the PS (Fig. 2). The PS, which represent the largest share of the CEC of illite, and the corresponding cation exchange selectivity coefficients, thus account for both inner- and outer-sphere binding of Tl^+^ on planar surfaces.

For the muscovites Musc-A and Musc-B, the XAS data pointed to predominant Tl adsorption *via* inner-sphere complexation onto siloxane cavities (Fig. 7). In line with this spectroscopic observation, the limited extraction of Tl adsorbed onto muscovite by CaCl_2_ and the high log *K*_d_ and log *K*_c,Tl–Na_ values (Fig. 1b, e and h) reflected that Tl was strongly retained. The log *K*_c,Tl–Na_ of Musc-A and Musc-B (3.0 and 3.1 at 3.2 μM dissolved Tl) were much larger than the Tl–Na exchange selectivity coefficient for the PS (1.2) in the 3-site model of illite.^8^ Therefore, we speculate that in the 3-site cation exchange model for illite, the T2S in combination with the PS also serve to account for variations in the adsorption affinity of Tl on planar surfaces as a function of surface loading, as different types of siloxane cavities^62^ get successively saturated.

In order to more rigorously test the ability of the 3-site cation exchange model to describe competitive cation adsorption onto structurally distinct types of sites on illite or other micaceous minerals, competitive adsorption experiments with two strongly adsorbing cations (*e.g.*, Tl and Cs or Rb) in mixed background electrolytes (*e.g.*, Ca and K) in combination with the spectroscopic characterization of the adsorbed ions could prove useful.

### 3.5. Thallium uptake by a soil clay mineral assemblage

#### Geogenic Tl in soil clay mineral assemblage

Only a minor fraction of the geogenic Tl of the Erzmatt topsoils from which the SCF was derived could be extracted as exchangeable Tl, although the XANES spectra of geogenic Tl resembled the spectrum of Tl adsorbed onto Na–IdP.^4,21^ It has therefore previously been suggested that most Tl may be fixed in the interlayers of micaceous clay minerals. In this study, both the XANES and EXAFS spectra of the geogenic Tl could be described by a minor fraction of the spectrum of Tl in the interlayer of Ba–muscovite and a major fraction of the spectrum of Tl adsorbed onto Na–IdP (Table 2; Fig. 7).^21^ This spectroscopic observation may indicate that Tl in the interlayers of micaceous minerals of the Erzmatt soils exhibits a lower short-range order than Tl incorporated into well-crystallized Ba–muscovite. Alternatively, the results could indicate that a major share of the operationally-defined “non-exchangeable” Tl could be retained in the collapsed interlayer zone at illite particle edges, with a local coordination similar to Tl freshly adsorbed in this zone. Consequently, the “non-exchangeability” of this Tl pool in 1 M NH_4_-acetate over a 1 to 1.5 h extraction period could be caused by slow exchange kinetics from the collapsed particle edges, whose decollapse and Tl release could be also hindered by the high NH_4_^+^ concentration in the extract, as has been shown for Cs.^64^ Studies on the adsorption and desorption kinetics of Cs^+^ on illite indeed revealed a gradual fixation of Cs in less rapidly exchangeable form over time, but also showed that even this fixed Cs remains exchangeable over sufficiently long equilibration periods.^64–66^

#### Freshly adsorbed Tl

Regarding the freshly adsorbed Tl, the isotherm data for the SCF revealed adsorption characteristics between those of Na–IdP and the muscovites Musc-A and Musc-B (Fig. 1). These macroscopic trends nicely matched with the spectroscopic results, which point to a shift from predominant Tl adsorption onto illite at the lower tested loadings to Tl adsorption onto muscovite at the higher loadings (Fig. 8, Table 2). Adsorption of Tl onto (collapsed) vermiculite appeared not to be relevant in the SCF. Due to the lack of a reference XAS spectrum for Tl adsorbed onto hydroxy-interlayered vermiculite (HIV), our results do not allow to conclude on the potential relevance of this uptake mechanism. For Cs, studies on its retention in soils with contrasting clay mineralogies suggest that HIV can bind Cs,^67,68^ but that HIV may be a less effective adsorbent than illite or weathered mica.^69^ We therefore assume that in the studied SCF (and more generally in the soils from which the SCF was derived^21^), Tl uptake by HIV was of minor relevance compared to uptake by illite and muscovite. As for Cs, however, HIV may be an important adsorbent for Tl in soils and sediments rich in HIV.

## 4. Conclusions

This study confirmed that micaceous clay minerals adsorb Tl through strong complexation at wedge sites in interlayers and through selective binding on planar surfaces. Variations in the extent and mode of Tl adsorption onto the different phyllosilicate minerals closely agree with trends reported for Cs and Rb. Accordingly, concepts for the assessment of the binding and solubility of radiocesium in soils and sediments such as the determination of the radiocesium interception potential (RIP) and considerations on adsorption competition with K and NH_4_, on adsorption reversibility,^66,70^ and on Cs availability for plant uptake^71^ should be transferable to Tl. Conversely, mechanistic insights into the adsorption of Tl(i) onto phyllosilicate minerals should equally be transferable to Cs and Rb.

In this study, the adsorption of Tl onto phyllosilicate minerals was examined over periods of 7 to 14 days. The kinetics of adsorption or the extent to which Tl exchangeability decreases over time through slow diffusion into collapsed interlayers or through trapping at collapsed particle edges was not addressed. Further work on these processes and their consequences for Tl retention and solubility can be based on available knowledge on Cs. Another factor that warrants further study is the pH-dependence of Tl adsorption onto the different clay minerals, including the potential adsorption of Tl at amphoteric edge sites at particle edges. Finally, relatively high Tl loadings (>200 mg kg^−1^) were employed in this work to obtain samples that could be probed by XAS. Very small fractions of highest affinity adsorption sites that could exist on all studied minerals and be relevant at lower Tl loadings may therefore have been overlooked. Considering that natural soils often contain <1 mg kg^−1^ Tl,^72^ and that even Tl contents in heavily contaminated soils and sediments rarely exceed 100 mg kg^−1^, the adsorption of Tl onto phyllosilicate minerals at trace levels, and in the presence of competing trace (Cs, Rb) and major cations (K, NH_4_) warrants further study.

## Conflicts of interest

There are no conflicts to declare.

## Supplementary Material

EM-024-D2EM00028H-s001
