# Supplementary material for: Thallium adsorption onto phyllosilicate minerals
Source: Environ Sci Process Impacts. 2022 May 24;24(9):1343–59. doi: 10.1039/d2em00028h (PMC9491347; doi:10.1039/d2em00028h)
Supplement: EM-024-D2EM00028H-s001 [file EM-024-D2EM00028H-s001.pdf]

Electronic Supplementary Material (ESI) for Environmental Science: Processes & Impacts.
This journal is © The Royal Society of Chemistry 2022

Electronic supplementary information

Thallium adsorption onto phyllosilicate minerals

Andreas Voegelin[1,], Silvan Wick[1], Numa Pfenninger[1], Stefan Mangold[2], Bart Baeyens[3], Maria Marques Fernandes[3]*

[1] Eawag, Swiss Federal Institute of Aquatic Science and Technology, Ueberlandstrasse 133, CH-8600 Duebendorf, Switzerland

[2] Karlsruhe Institute of Technology, Hermann-von-Helmholtz Platz 1, D-76344 Eggenstein-Leopoldshafen, Germany

[3] Paul Scherrer Institute, Forschungsstrasse 111, CH-5232 Villigen PSI, Switzerland

[*] Correspondence: andreas.voegelin@eawag.ch

Contents

1. Sample characterization 2
   1.1. X-ray diffraction (XRD) 2
   1.2. Scanning electron microscopy - energy-dispersive X-ray (SEM-EDX) analyses 4
   1.3. Cation exchange capacity (CEC) determinations 8
2. Sorption data 9
3. X-ray absorption spectroscopy 11
   3.1. Overview over XAS samples and measurements 11
   3.2. Tl L_{III}-edge XAS of Tl(III) and Tl(I) reference compounds 11
   3.3. Tl L_{III}-edge XANES spectra of Tl-sorbed clay minerals 15
   3.4. LCF analysis of XANES spectra of Tl-loaded vermiculites 21
   3.5. Shell-fit analysis of selected EXAFS spectra 22
4. References 30

1. Sample characterization

1.1. X-ray diffraction (XRD)

For analysis by X-ray diffraction, about 20 mg of the samples Musc-A, Musc-B, Verm-A and Verm-B were suspended in ethanol, deposited on low-background Si wafers and air-dried. For Musc-A, Musc-B, and Verm-A, sample aliquots as prepared for adsorption experiments were used, for Verm-B, an unmilled sample aliquot was used. XRD patterns were recorded from 5° to 95° 2-theta (step-size of 0.017°, 2 or 5.5 h per pattern, Co Kα radiation, X'Pert Powder diffractometer with XCelerator detector, Malvern Panalytical B.V., Almelo, The Netherlands).

The patterns of the muscovites and vermiculites are shown in Fig. S1. Qualitatively, they matched to muscovite and vermiculite reference patterns from the Crystallography Open Database (COD) (not shown), confirming phase identity. For the vermiculites, spectral differences between Verm-A (milled) and Verm-B (unmilled) were in line with the reported effect of milling on the XRD pattern of vermiculite.[1]

For XRD analysis, the SCF (soil clay fraction) was exchanged with Mg or K and prepared as oriented mounts on low-background Si slides. After measurement at room temperature, the Mg-exchanged SCF was reacted with ethylene glycol (5.5 h at 60°C) and analyzed again. The K-exchanged SCF was heated to temperatures of 100, 300 and 550°C (1 h). After each heat treatment, the sample was allowed to cool and an XRD pattern was recorded.

The XRD patterns of the oriented mounts are shown in Fig. S2. The pattern of the Mg-exchanged SCF indicated illite/muscovite (10-Å peak; ~10.2°), hydroxy-interlayered vermiculite (broad peak between 10- and 14-Å peaks), and smectite or vermiculite (14-Å peak; 7.2°). EG treatment caused some interlayer expansion (lower-angle shoulder in the 14-Å peak at ~7.2°), indicative of the presence of smectite. K-exchange induced the (partial) shift of the 14-Å (7.2°) peak to 10 Å (10.2°), in line with the presence of (hydrox-interlayered) vermiculite. Heating to 550°C caused the disappearance of the 7-Å (14.3°) peak, in line with the destruction of kaolinite and only a minor fraction of chlorite. Overall, these results are in good agreement with earlier XRD results for the clay fraction of the soil P1 00-20 from which the SCF was isolated, which indicated that illite/muscovite and hydroxy-interlayered-vermiculite/chlorite were the dominant phylloscilicates, with minor fractions of illite-smectite and some kaolinite.[2]

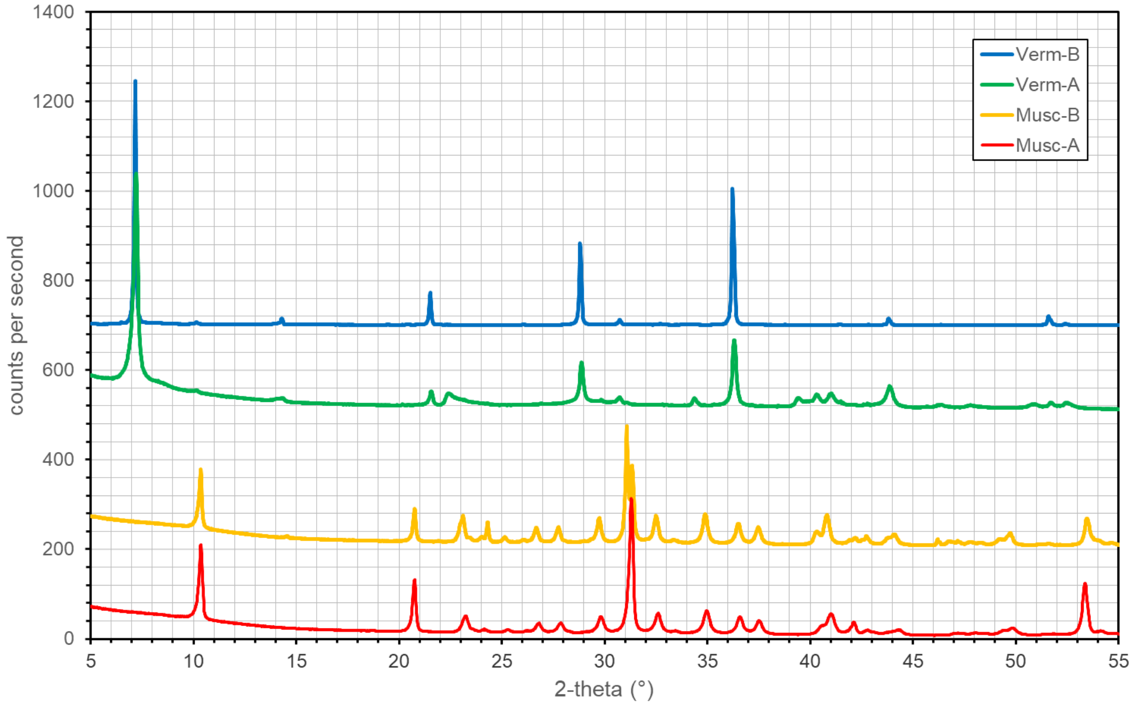

Fig. S1. XRD patterns of the samples Musc-A, Musc-B, Verm-A and Verm-B (unmilled) measured with Co Kα radiation. The patterns are stacked (Musc-B: + 200, Verm-A: +500, Verm-B: +700) and the pattern of sample Verm-B scaled by 0.05 for better comparison.

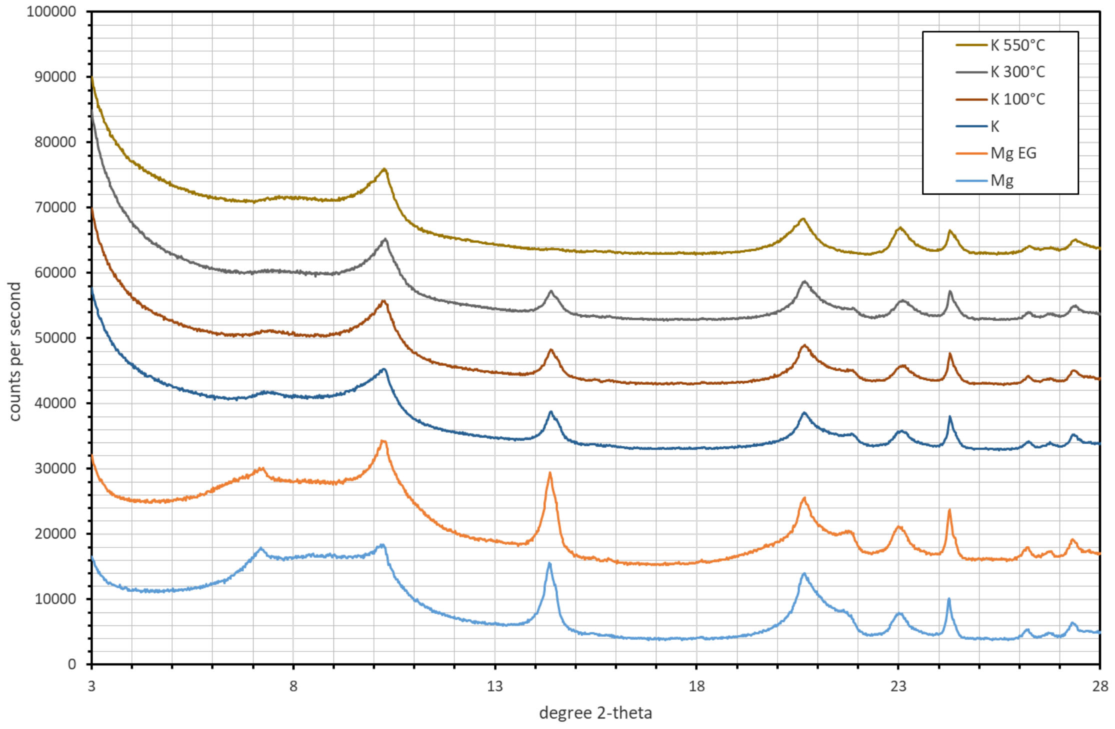

Fig. S2. XRD patterns of oriented mounts of the Mg- and K-exchanged soil clay fraction (SCF), including patterns of ethylene glycol treatment of the Mg-exchanged SCF and heat treatment of the K-exchanged SCF (measured with Co Kα radiation).

1.2. Scanning electron microscopy - energy-dispersive X-ray (SEM-EDX) analyses

For SEM-EDX analyses, 2 mg aliquots of the samples Musc-A 870, Musc-B 860, Verm-A 820 and Verm-B 630 (numbers refer to Tl loading in mg/kg) were dispersed in 10 mL of doubly deionized (DDI) water, subsequently deposited onto Nucleopore filters by vacuum filtration, dried, and C-coated (Leica ACE600). The soil clay fraction was analogously prepared after diluting 50 µL of the SCF suspension (with 53 g/L solids) into 10 mL DDI water. The SEM (NanoSEM 230, FEI) was operated at an acceleration voltage of 15 kV. EDX data (X-MAX 80 detector, Oxford Instruments) were collected and quantitatively evaluated using the INCA software package (Version 4.15, Oxford Instruments).

Backscattered electron (BSE) images of the muscovites and vermiculites are shown in Fig. S3, the results from EDX point analyses are provided in Table S1. All samples exhibited the typical platy morphology of phyllosilicate minerals. For the muscovites, the data pointed to a larger share of finer material in the Musc-B sample. For the vermiculites, the results revealed that the sample Verm-A contained a much larger share of fine material than in Verm-A, and showed that larger vermiculite platelets dominated in Verm-B. The elemental composition data was in line with expectations for muscovites (high K and Al, low Mg) and vermiculites (high Mg and Fe, low K).

BSE images of SCF are shown in Fig. S4, the results from EDX analyses on a range of points are listed in Table S2. Both sample morphology and EDX results attest to the heterogeneity of the soil clay fraction. The EDX results could be associated to 5 major groups based on element abundances (probable mineral phase association): (i) high Si (quartz), (ii) high Ti (anatase), (iii) high K / low Mg & Fe (illite, muscovite), (iv) low K / high Mg and Fe (chlorite), (v) intermediate K, Mg & Fe / high Al (hydroxy-interlayered vermiculite). The probable mineral phase associations are based on earlier XRD results for the soil P1 00-20[2] from which the SCF was isolated, and on XRD results for the SCF shown in the electronic supplementary information (ESI) section 1.1.

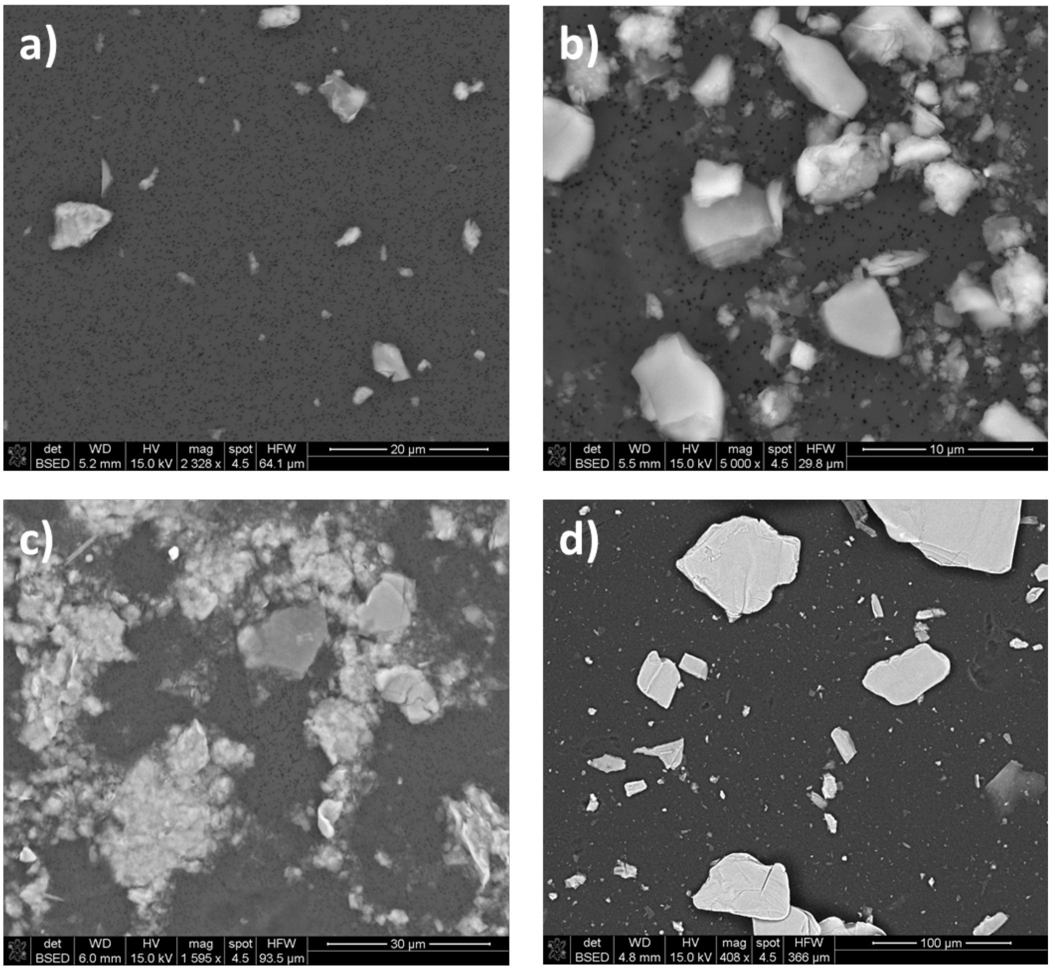

Fig. S3. Backscattered electron images of the samples (a) Musc-A 780, (b) Musc-B 860, (c) Verm-A 820 and (d) Verm-B 630.

Table S1. Elemental composition of the samples Musc-A, Musc-B, Verm-A and Verm-B as derived from multipoint SEM-EDX analyses (n: number of points; mean and standard deviation (stdev); values represent normalized weight fractions). The content of O was calculated based on the stoichiometry of the metal oxides, assuming Fe to be bivalent (FeO).

	**n**		**Na**	**Mg**	**Al**	**Si**	**K**	**Ca**	**Ti**	**Fe**	**O**
**Musc-A**	13	mean	0.6	0.3	18.4	23.1	9.6	0.0	0.2	2.1	45.7
		stdev	*0.2*	*0.1*	*0.6*	*0.5*	*1.0*	*0.2*	*0.3*	*0.7*	*0.5*
**Musc-B**	11	mean	0.2	1.9	14.0	25.9	8.3	0.0	0.2	3.2	46.2
		stdev	*0.2*	*0.3*	*0.7*	*1.3*	*1.6*	*0.1*	*0.2*	*0.8*	*0.8*
**Verm-A**	13	mean	1.9	17.1	6.6	21.4	0.2	0.5	0.3	7.2	44.7
		stdev	*0.5*	*1.4*	*1.4*	*0.3*	*0.2*	*0.2*	*0.2*	*0.8*	*0.3*
**Verm-B**	8	mean	3.1	16.5	7.6	21.0	0.0	0.3	0.8	5.8	44.9
		stdev	*1.8*	*1.0*	*0.7*	*1.4*	*0.1*	*0.1*	*0.2*	*0.8*	*0.8*

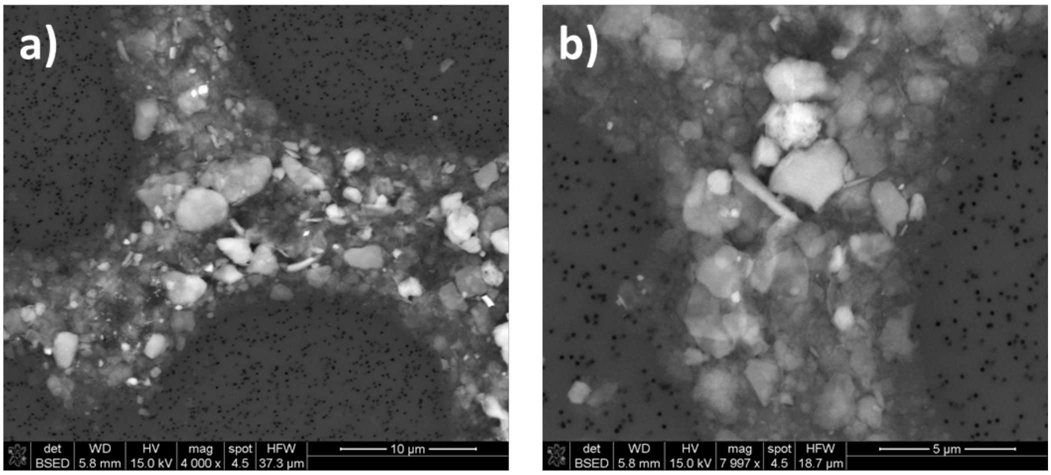

Fig. S4. Backscattered electron images of the soil clay fraction SCF.

Table S2. Elemental composition data collected on the soil clay fraction SCF, grouped according to element abundances (values represent normalized weight fractions). The content of O was calculated based on the stoichiometry of the metal oxides, assuming Fe to be bivalent (FeO).

	**Na**	**Mg**	**Al**	**Si**	**K**	**Ca**	**Ti**	**Fe**	**O**
**high Si**									
**S3.5**	0.1	0.3	1.6	44.5	0.4	0.0	0.0	0.3	52.6
**S3.2**	0.3	0.4	1.9	43.8	0.5	0.1	0.0	0.9	52.2
**S2.3**	0.1	0.4	2.5	43.5	0.8	-0.1	0.0	0.4	52.3
**S4.2**	0.2	0.8	3.4	41.2	1.3	0.1	0.0	1.5	51.4
**high Ti**									
**S2.5**	0.0	0.6	2.9	4.4	0.9	-0.1	49.2	0.9	41.2
**S2.6**	0.0	0.6	2.9	6.5	1.4	0.0	45.5	1.5	41.5
**S2.7**	0.4	0.8	5.0	9.7	2.7	-0.1	38.0	1.2	42.4
S3.7	*0.5*	*2.4*	*3.5*	*5.4*	*0.4*	*-0.1*	*28.7*	*22.5*	*36.7*
**high K - low Mg**									
**S3.6**	0.1	0.7	9.7	30.4	11.5	0.0	0.3	0.8	46.5
**S1.1**	0.3	1.1	10.2	28.6	10.8	0.1	0.4	2.7	45.8
**S4.3**	0.3	0.9	9.8	30.1	10.8	0.0	0.0	1.5	46.4
**S4.1**	0.2	0.6	9.1	31.7	10.5	0.1	0.2	0.5	47.1
**S2.2**	0.1	1.0	9.5	31.0	10.2	0.0	0.1	1.1	47.0
**low K - high Mg & Fe**									
**S2.2**	0.6	5.3	11.9	16.9	0.6	0.2	0.0	24.1	40.6
**S1.2**	0.3	7.5	11.8	17.4	1.0	0.0	0.2	20.2	41.6
**S3.1**	0.2	6.6	11.3	19.8	1.8	-0.4	-0.1	18.3	42.4
**S1.3**	0.3	10.8	12.0	17.4	0.5	0.2	-0.1	16.3	42.5
**S2.10**	0.6	4.7	11.7	21.8	2.3	0.3	-0.1	15.3	43.4
**intermediate K & Fe / high Al - sorted by decreasing K**									
**S4.5**	0.2	2.0	14.2	25.2	7.1	0.1	0.5	4.5	46.0
**S2.2**	0.7	1.0	15.6	24.9	5.7	0.2	0.7	5.0	46.2
**S4.6**	0.4	1.4	16.1	25.8	5.6	0.0	0.1	3.6	47.0
**S1.4**	0.8	1.3	16.5	25.8	5.3	0.0	0.2	2.9	47.3
**S1.5**	0.2	2.2	14.5	27.5	4.8	-0.2	0.2	3.1	47.7
**S3.4**	0.3	1.7	15.2	28.1	4.4	0.0	0.1	1.8	48.3
**S3.8**	0.8	1.9	14.9	27.1	3.5	0.3	0.6	3.2	47.8
**S3.3**	3.0	0.8	15.9	27.4	3.2	0.0	0.4	1.1	48.2
**S4.4**	0.7	1.4	6.8	36.1	2.4	0.0	0.3	2.5	49.8
**S2.9**	0.2	1.8	15.3	26.6	2.2	0.1	0.1	6.3	47.5
**S2.4**	3.3	0.5	17.8	26.5	1.5	0.3	0.4	1.3	48.5

1.3. Cation exchange capacity (CEC) determinations

The CEC of the different solids listed in Table 1 were obtained by different methods:

For the illite IdP, a CEC of 263 $mmol_c$/kg had previously been identified for Cs-exchanged IdP using the ^{137}Cs isotope exchange technique,[3] and a CEC of 260 $mmol_c$/kg for Tl-exchanged IdP using the same isotope exchange approach with ^{204}Tl instead of ^{137}Cs as radioisotope.[4] The CEC of 260 mol_c/kg was taken for Na-IdP and K-IdP.

For the smectite SWy used in this study, a CEC of 860 $mmol_c$/kg has been determined using the ^{137}Cs isotope technique. For similar types of smectites, a CEC of 870 $mmol_c$/kg has previously been determined using ^{22}Na isotope dilution,[5] and a CEC of 0.89 $mmol_c$/kg using Cs/K exchange.[6]

For the two muscovites, the ^{137}Cs isotope technique was used to measure the CEC on the residual solids from the Tl adsorption experiments with ~1000 mg/kg adsorbed Tl (as the available material was limited). The measurements returned CEC values of 121 $mmol_c$/kg for Musc-A and 145 $mmol_c$/kg for Musc-B. These values were single determinations. They fall into the range of CEC values of muscovites reported in the literature.[7, 8]

For the vermiculite Verm-A, a CEC of 1320 $mmol_c$/kg was determined by Ca-Mg followed by Ba-Ca exchange. Briefly, the vermiculite was first twice saturated with 100 mM Mg, then twice washed with doubly deionized water. Subsequently, the vermiculite was saturated twice with 100 mM Ca. The Mg released into the saturation solutions was analyzed by inductively coupled plasma - mass spectrometry (ICP-MS). The sample was then washed twice with DDI water, before being saturated twice with 100 mM Ba. The Ca released during Ba saturation was also analyzed by ICP-MS. The reported CEC corresponds to the average of the CEC values calculated from Ca-Mg (1280 $mmol_c$/kg) and Ba-Ca (1360 $mmol_c$/kg) exchange. For the vermiculite Verm-B, a CEC of 1590 $mmol_c$/kg has been reported for raw material from the same source[1] based on the ammonium acetate method.[9] The CEC values for both Verm-A and Verm-B fall into the common range of CEC values of trioctahedral vermiculites.[7]

For SCF, a CEC of 290 $mmol_c$/kg was determined using the ^{137}Cs isotope exchange technique,[3] A similar value had been obtained by Ca-Mg / Ba-Ca exchange (as described for Verm-A above).

2. Sorption data

Table S3. Adsorption data: mineral name, sample name as used in figures and text (mineral followed by Tl loading in mg/kg), suspension volume, mineral mass, total Tl in suspension, equilibration time, final pH, final dissolved Tl (C_Tl) and final adsorbed Tl loading (Q_Tl), adsorbed Tl equivalent fraction (N_{Tl}), distribution coefficient ($\log(K_d/(L/kg))$) and conditional Tl-Na cation exchange selectivity coefficient ($\log(K_{c,Tl\text{-}Na})$; not applicable to K-IdP, as adsorption was m in 10 mM KCl electrolyte).

clay mineral	sample name	volume	mass	tot_Tl	time	pH	C_Tl	Q_Tl	Q_Tl	N_Tl	log K_d	log ($K_{c,Tl\text{-}Na}$)
		(mL)	*(mg)*	*(mol/L)*	*(d)*		*(mol/L)*	*(mol/kg)*	*(mg/kg)*	*(-)*	*(L/kg)*	*(-)*
Na-IdP	Na-IdP 350	35	154	7.83E-06	~14	7.1	2.56E-07	1.72E-03	350	6.61E-03	3.83	3.42
Na-IdP	Na-IdP 1100	35	154	2.61E-05	~14	6.9	1.51E-06	5.58E-03	1138	2.15E-02	3.57	3.16
Na-IdP	Na-IdP 3200	35	154	7.83E-05	~14	6.5	1.01E-05	1.55E-02	3156	5.96E-02	3.19	2.80
Na-IdP	Na-IdP 8000	35	154	2.54E-04	~14	7.1	8.15E-05	3.92E-02	7998	1.51E-01	2.68	2.34
Na-IdP	Na-IdP 13000	35	154	7.64E-04	~14	6.9	4.75E-04	6.55E-02	13350	2.52E-01	2.14	1.85
K-IdP	K-IdP 170	35	142	7.83E-06	~14	6.6	4.49E-06	8.23E-04	168	3.16E-03	2.26	-
K-IdP	K-IdP 500	35	142	2.61E-05	~14	6.6	1.61E-05	2.46E-03	501	9.46E-03	2.18	-
K-IdP	K-IdP 1400	35	142	7.81E-05	~14	6.5	5.09E-05	6.69E-03	1364	2.57E-02	2.12	-
K-IdP	K-IdP 4200	35	142	2.51E-04	~14	6.5	1.69E-04	2.05E-02	4171	7.87E-02	2.08	-
K-IdP	K-IdP 9700	36	142	7.39E-04	~14	6.5	5.52E-04	4.75E-02	9685	1.83E-01	1.94	-
Na-SWy	Na-SWy 110	35	201	7.83E-06	~14	7.9	4.83E-06	5.24E-04	107	6.10E-04	2.04	1.10
Na-SWy	Na-SWy 270	35	201	2.61E-05	~14	6.9	1.87E-05	1.30E-03	265	1.51E-03	1.84	0.91
Na-SWy	Na-SWy 740	35	201	7.83E-05	~14	6.9	5.73E-05	3.65E-03	744	4.25E-03	1.80	0.87
Na-SWy	Na-SWy 1700	35	201	2.54E-04	~14	6.8	2.05E-04	8.52E-03	1737	9.92E-03	1.61	0.69
Na-SWy	Na-SWy 5200	35	201	7.64E-04	~14	6.8	6.18E-04	2.54E-02	5173	2.95E-02	1.61	0.69
SCF	SCF 390	40	247	1.30E-05	7	6.6	1.02E-06	1.92E-03	392	6.62E-03	3.27	2.81
SCF	SCF 760	40	247	2.59E-05	7	6.5	2.68E-06	3.74E-03	764	1.29E-02	3.14	2.69
SCF	SCF 1800	40	247	6.45E-05	7	6.5	1.11E-05	8.62E-03	1762	2.97E-02	2.89	2.44
SCF	SCF 5100	41	247	2.29E-04	7	6.4	7.74E-05	2.48E-02	5070	8.55E-02	2.51	2.08
SCF	SCF 10000	42	247	6.11E-04	7	6.3	3.14E-04	5.06E-02	10338	1.74E-01	2.21	1.83

Table S3. Continuation.

clay mineral	sample name	volume	mass	tot_Tl	time	pH	C_Tl	Q_Tl	Q_Tl	N_Tl	log K_d	log ($K_{c,Tl\text{-}Na}$)
		(mL)	*(mg)*	*(mol/L)*	*(d)*		*(mol/L)*	*(mol/kg)*	*(mg/kg)*	*(-)*	*(L/kg)*	*(-)*
Musc-A	Musc-A 300	35	198	8.94E-06	7	6.6	7.73E-07	1.45E-03	297	1.20E-02	3.83	3.20
Musc-A	Musc-A 870	35	198	2.96E-05	7	6.8	5.82E-06	4.25E-03	868	3.51E-02	3.57	2.80
Musc-A	Musc-A 2400	36	202	8.67E-05	7	7.0	2.10E-05	1.18E-02	2411	9.75E-02	3.19	2.71
Musc-A	Musc-A 6400	39	200	2.68E-04	7	6.5	1.08E-04	3.13E-02	6397	2.59E-01	2.68	2.51
Musc-A	Musc-A 12000	47	201	6.55E-04	7	6.5	4.01E-04	5.93E-02	12129	4.90E-01	2.14	2.38
Musc-B	Musc-B 250	26	149	7.35E-06	7	7.8	3.56E-07	1.22E-03	250	8.49E-03	2.26	3.38
Musc-B	Musc-B 860	26	150	2.57E-05	7	7.9	1.62E-06	4.19E-03	856	2.91E-02	2.18	3.27
Musc-B	Musc-B 2600	26	150	8.03E-05	7	7.9	8.07E-06	1.26E-02	2581	8.77E-02	2.12	3.08
Musc-B	Musc-B 7400	27	150	2.65E-04	7	7.9	6.25E-05	3.61E-02	7384	2.51E-01	2.08	2.73
Musc-B	Musc-B 16000	28	150	7.46E-04	7	7.8	3.19E-04	8.04E-02	16422	5.58E-01	1.94	2.60
Verm-A	Verm-A 210	35	202	8.94E-06	7	7.9	3.14E-06	1.01E-03	206	7.65E-04	2.04	1.39
Verm-A	Verm-A 820	35	200	2.96E-05	7	8.3	6.98E-06	3.99E-03	815	3.02E-03	1.84	1.64
Verm-A	Verm-A 2700	36	201	8.67E-05	7	8.2	1.40E-05	1.31E-02	2676	9.92E-03	1.80	1.86
Verm-A	Verm-A 8900	39	202	2.68E-04	7	8.5	4.25E-05	4.37E-02	8933	3.31E-02	1.61	1.91
Verm-A	Verm-A 28000	47	200	6.55E-04	7	8.6	6.67E-05	1.37E-01	28035	1.04E-01	1.61	2.24
Verm-B	Verm-B 170	26	109	7.35E-06	7	8.4	3.82E-06	8.46E-04	173	5.32E-04	3.27	1.14
Verm-B	Verm-B 630	26	151	2.57E-05	7	8.4	7.77E-06	3.10E-03	633	1.95E-03	3.14	1.40
Verm-B	Verm-B 2400	26	151	8.03E-05	7	8.5	1.35E-05	1.16E-02	2370	7.29E-03	2.89	1.74
Verm-B	Verm-B 9100	27	150	2.65E-04	7	8.6	1.45E-05	4.45E-02	9093	2.80E-02	2.51	2.30
Verm-B	Verm-B 28000	28	149	7.46E-04	7	8.2	1.77E-05	1.38E-01	28203	8.68E-02	2.21	2.73

3. X-ray absorption spectroscopy

3.1. Overview over XAS samples and measurements

An overview over the XAS samples and measurements used in this study is given in Table S4. Most samples were Tl-loaded solids from the adsorption experiments of this study, few samples were from own earlier work.[4, 10] The sample names of Tl-loaded phyllosilicates consist of the mineral name followed by the Tl loading in mg/kg. The XAS measurement type (XANES, EXAFS) is indicated together with measurement temperature in parentheses (RT = room temperature, 20 K = 20 Kelvin). Further details on the XAS measurements are given in the Materials and Methods section of the manuscript.

3.2. Tl L_{III}-edge XAS of Tl(III) and Tl(I) reference compounds

In Fig. S5, the XANES and EXAFS spectra of Ba-muscovite and aqueous Tl^+, both monovalent Tl(I) compounds, are compared to the spectrum of Tl_2O_3, trivalent Tl(III)-oxide. The XANES spectrum of Tl_2O_3 features a pre-edge shoulder at ~12655 eV and a peak at 12687 eV that are characteristic for Tl(III) and absent in the spectra of Tl(I) compounds. The oscillations in the EXAFS spectrum of Tl_2O_3 up to ~7 $Å^{-1}$ mainly arise from first-shell Tl-O contributions of regularly ordered TlO_6 octahedra in Tl_2O_3, as reflected by the large first-shell Tl-O peak in the corresponding FT EXAFS spectrum (Fig. S5c). In contrast, the first-shell Tl-O signal of the Tl(I) compounds is strongly attenuated by a high degree of disorder. Consequently, the EXAFS of the Ba-muscovite is dominated by the second-shell Tl-Si peak (see ESI section 3.4). Owing to the characteristic XANES and EXAFS features of Tl(III) (in Tl_2O_3) as compared to Tl(I) (aqueous Tl^+ and Ba-muscovite), spectral contributions of more than 5-10% Tl(III) should have been detectable in the XANES and EXAFS spectra of the Tl-loaded phyllosilicate samples.

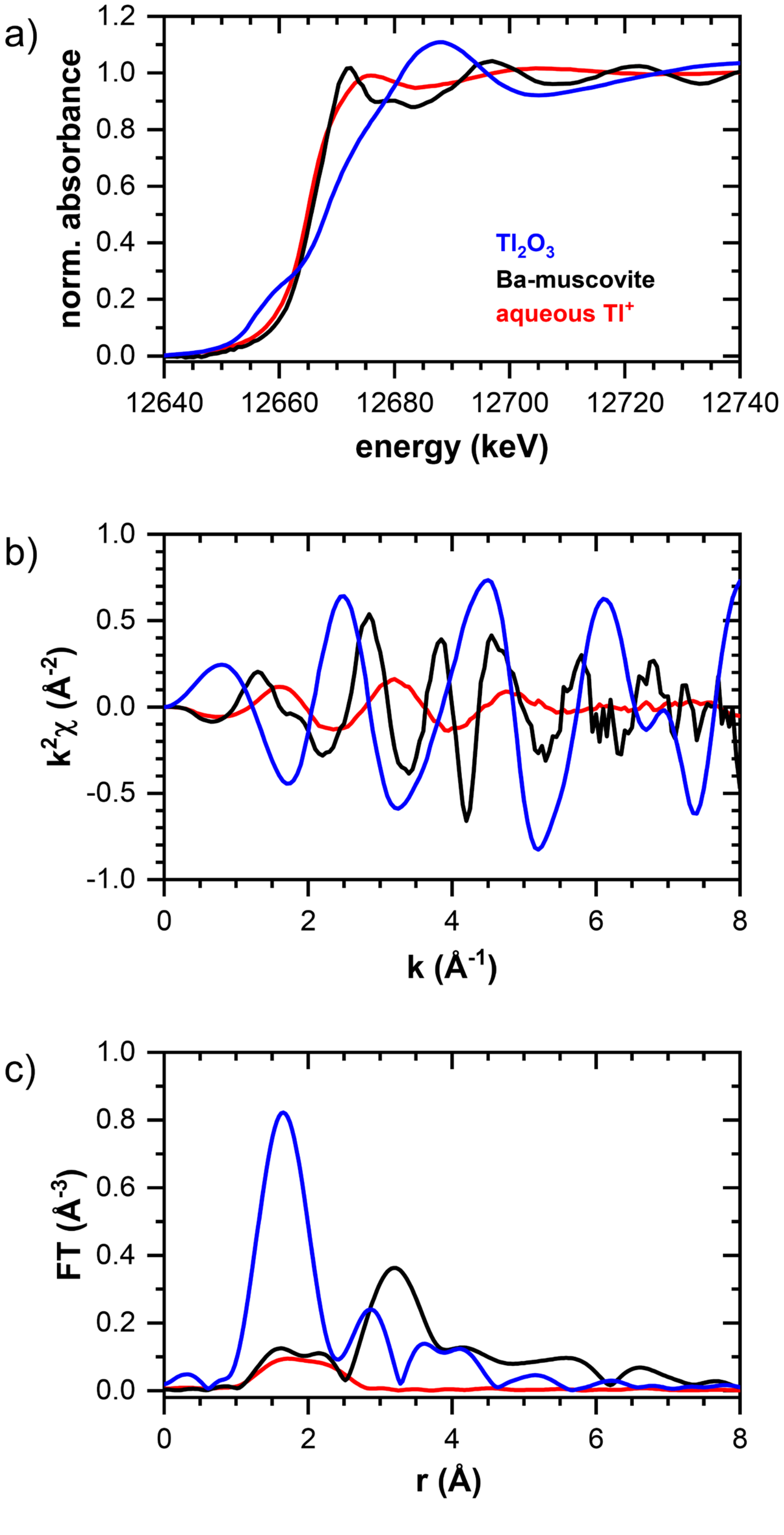

Fig. S5. Tl L_{III}-edge XAS spectra of trivalent Tl in Tl_2O_3 (from Voegelin et al. (2015)[2]) and monovalent Tl in Ba-muscovite and as aqueous Tl^+. (a) XANES spectra, (b) k^2-weighted EXAFS, spectra and (c) magnitude of the Fourier-transform of the EXAFS spectra.

Table S4. Spectroscopy data: mineral name, XAS sample name (for Tl-loaded phyllosilicates: mineral name followed by Tl loading in mg/kg), measurement type, and beamline used.

mineral	sample name	sample origin	comment	measurement	beamline
Na-IdP	Na-IdP 350	this study	adsorbed Tl	XANES (RT)	SAMBA (Soleil)
	Na-IdP 1100	this study	adsorbed Tl	XANES (RT)	SAMBA (Soleil)
	Na-IdP 3200	this study	adsorbed Tl	XANES (RT) + EXAFS (20 K)	SAMBA (Soleil)
	Na-IdP 8000	this study	adsorbed Tl	XANES (RT)	SAMBA (Soleil)
	Na-IdP 13000	this study	adsorbed Tl	XANES (RT)	SAMBA (Soleil)
	Na-IdP 50	Wick et al. (2018)	adsorbed Tl	XANES (RT) + EXAFS (RT)	SuperXAS (SLS)
	Na-IdP 300	Wick et al. (2018)	adsorbed Tl	XANES (RT) + EXAFS (RT)	SuperXAS (SLS)
	Na-IdP 3800	Wick et al. (2018)	adsorbed Tl	XANES (RT) + EXAFS (RT)	SuperXAS (SLS)
K-IdP	K-IdP 170	this study	adsorbed Tl	not analyzed	
	K-IdP 500	this study	adsorbed Tl	XANES (RT)	XAS (KIT)
	K-IdP 1400	this study	adsorbed Tl	XANES (RT)	XAS (KIT)
	K-IdP 4200	this study	adsorbed Tl	XANES (RT) + EXAFS (RT)	XAS (KIT)
	K-IdP 9700	this study	adsorbed Tl	XANES (RT)	XAS (KIT)
Na-SWy	Na-SWy 110	this study	adsorbed Tl	not analyzed	
	Na-SWy 270	this study	adsorbed Tl	XANES (RT)	XAS (KIT)
	Na-SWy 740	this study	adsorbed Tl	XANES (RT)	XAS (KIT)
	Na-SWy 1700	this study	adsorbed Tl	XANES (RT) + EXAFS (RT)	XAS (KIT)
	Na-SWy 5200	this study	adsorbed Tl	XANES (RT)	XAS (KIT)
Musc-A	Musc-A 300	this study	adsorbed Tl	XANES (RT)	XAS (KIT)
	Musc-A 870	this study	adsorbed Tl	XANES (RT)	XAS (KIT)
	Musc-A 2400	this study	adsorbed Tl	XANES (RT) + EXAFS (RT)	XAS (KIT)
	Musc-A 6400	this study	adsorbed Tl	XANES (RT)	XAS (KIT)
	Musc-A 12000	this study	adsorbed Tl	XANES (RT)	XAS (KIT)

Table S4. Continuation.

mineral	sample name	sample origin	comment	measurement	beamline
Musc-B	Musc-B 250	this study	adsorbed Tl	XANES (RT)	SAMBA (Soleil)
	Musc-B 860	this study	adsorbed Tl	XANES (RT)	SAMBA (Soleil)
	Musc-B 2600	this study	adsorbed Tl	XANES (RT)	SAMBA (Soleil)
	Musc-B 7400	this study	adsorbed Tl	XANES (RT) + EXAFS (RT)	SAMBA (Soleil)
	Musc-B 16000	this study	adsorbed Tl	XANES (RT)	SAMBA (Soleil)
	Musc-B 16000 Ca ex	this study	after $CaCl_2$ extraction	XANES (RT)	SAMBA (Soleil)
Verm-A	Verm-A 210	this study	adsorbed Tl	XANES (RT)	XAS (KIT)
	Verm-A 820	this study	adsorbed Tl	XANES (RT)	XAS (KIT)
	Verm-A 2700	this study	adsorbed Tl	XANES (RT) + EXAFS (RT)	XAS (KIT)
	Verm-A 8900	this study	adsorbed Tl	XANES (RT)	XAS (KIT)
	Verm-A 28000	this study	adsorbed Tl	XANES (RT)	XAS (KIT)
Verm-B	Verm-B 170	this study	adsorbed Tl	XANES (RT)	SAMBA (Soleil)
	Verm-B 630	this study	adsorbed Tl	XANES (RT)	SAMBA (Soleil)
	Verm-B 2400	this study	adsorbed Tl	XANES (RT)	SAMBA (Soleil)
	Verm-B 9100	this study	adsorbed Tl	XANES (RT)	SAMBA (Soleil)
	Verm-B 28000	this study	adsorbed Tl	XANES (RT) + EXAFS (RT)	SAMBA (Soleil)
SCF	SCF geogenic	Wick et al. (2020)	natural Tl content	XANES (RT) + EXAFS (RT)	DUBBLE (ESRF)
	SCF 390	this study	adsorbed Tl	XANES (RT)	DUBBLE (ESRF)
	SCF 760	this study	adsorbed Tl	XANES (RT)	DUBBLE (ESRF)
	SCF 1800	this study	adsorbed Tl	XANES (RT) + EXAFS (RT)	DUBBLE (ESRF)
	SCF 5100	this study	adsorbed Tl	XANES (RT)	DUBBLE (ESRF)
	SCF 10000	this study	adsorbed Tl	XANES (RT) + EXAFS (RT)	DUBBLE (ESRF)
Ba-muscovite	Ba-muscovite	Wick et al. (2020)	natural Tl content	XANES (RT) + EXAFS (RT)	SuperXAS (SLS)
			natural Tl content	EXAFS (20 K)	SAMBA (Soleil)
10 mM $TlNO_3$	aqueous Tl^+	Wick et al. (2018)	aqueous solution	XANES (RT) + EXAFS (RT)	SuperXAS (SLS)
Tl_2O_3	Tl_2O_3	Voegelin et al. (2015)	Tl_2O_3 (avicennite)	XANES (RT) + EXAFS (RT)	XAS (KIT)

3.3. Tl L$_{III}$-edge XANES spectra of Tl-sorbed clay minerals

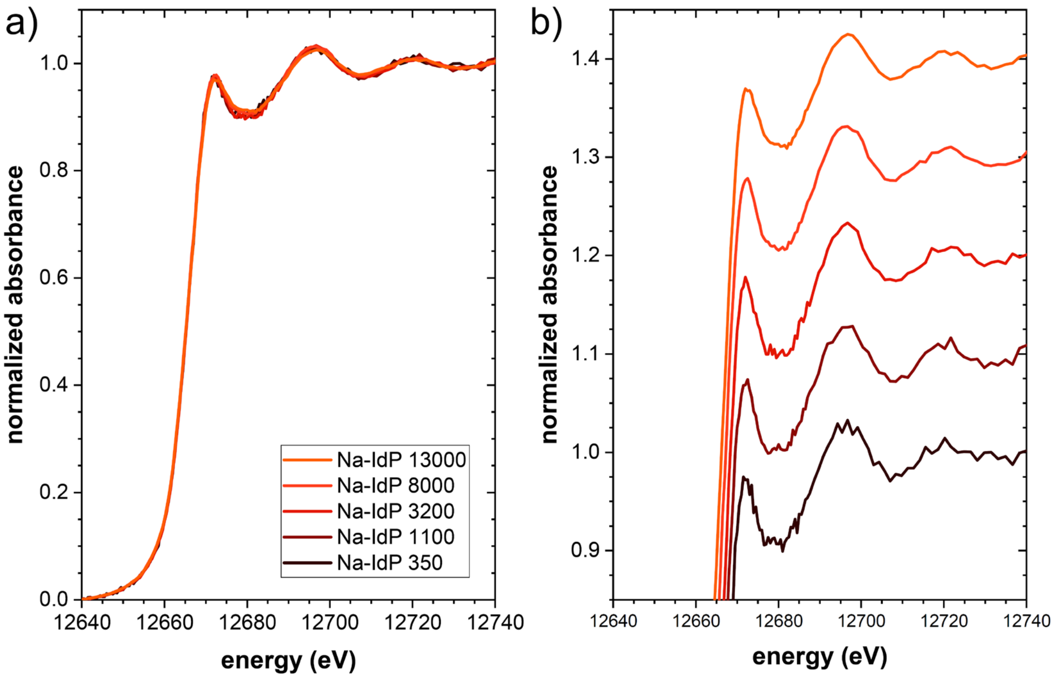

Fig. S6. XANES spectra of Tl adsorbed onto Na-IdP (numbers indicate Tl loading in mg/kg). (a) Overlay plot. (b) Zoom into stacked plot with spectra offset by offset of 0.1 between spectra.

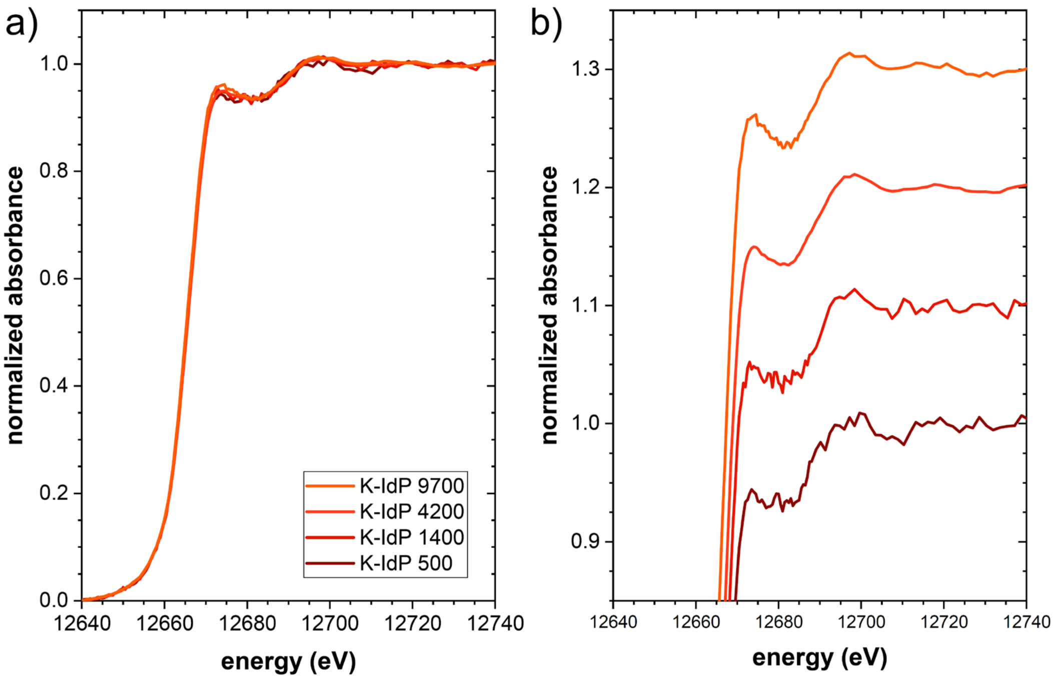

Fig. S7. XANES spectra of Tl adsorbed onto K-IdP (numbers indicate Tl loading in mg/kg). (a) Overlay plot. (b) Zoom into stacked plot with y-axis offset of 0.1 between spectra.

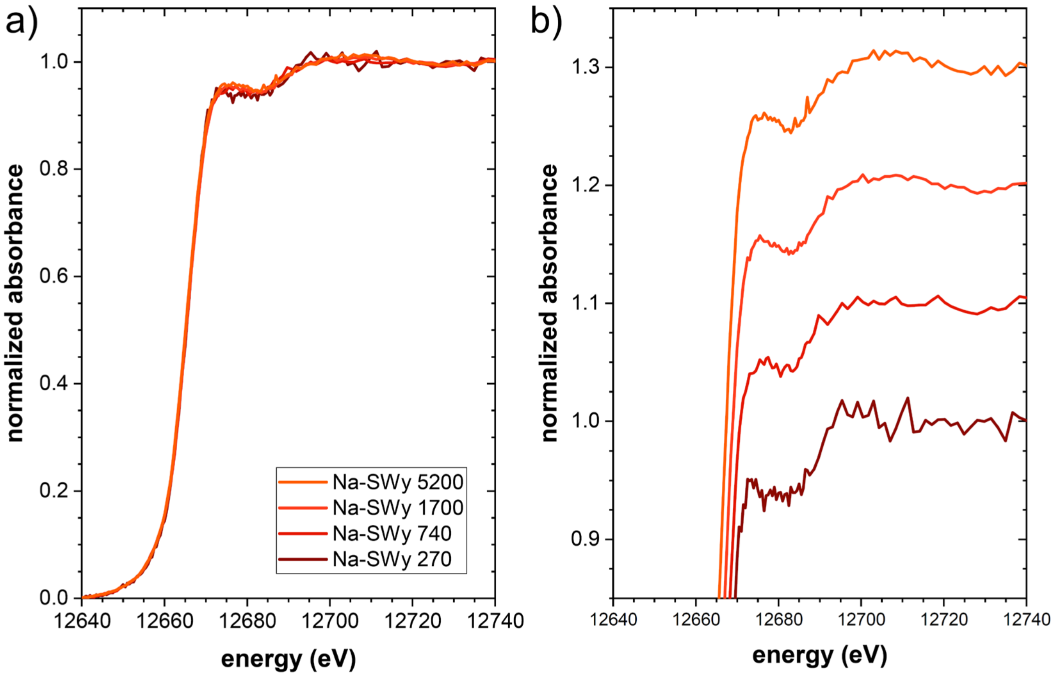

Fig. S8. XANES spectra of Tl adsorbed onto Na-SWy (numbers indicate Tl loading in mg/kg). (a) Overlay plot. (b) Zoom into stacked plot with y-axis offset of 0.1 between spectra.

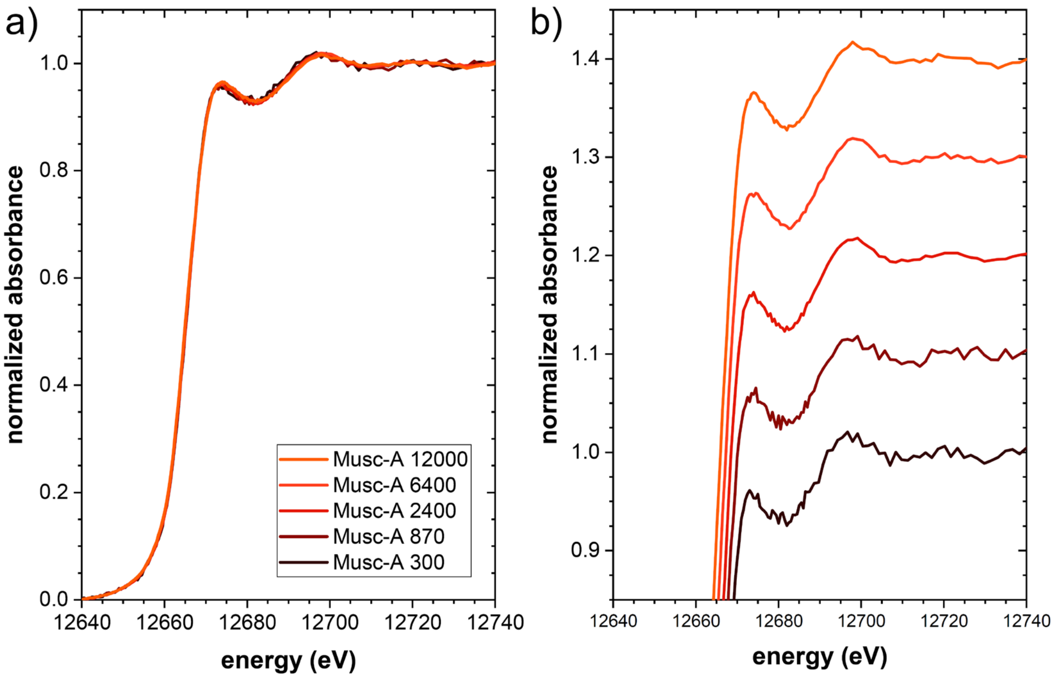

Fig. S9. XANES spectra of Tl adsorbed onto Musc-A (numbers indicate Tl loading in mg/kg). (a) Overlay plot. (b) Zoom into stacked plot with y-axis offset of 0.1 between spectra.

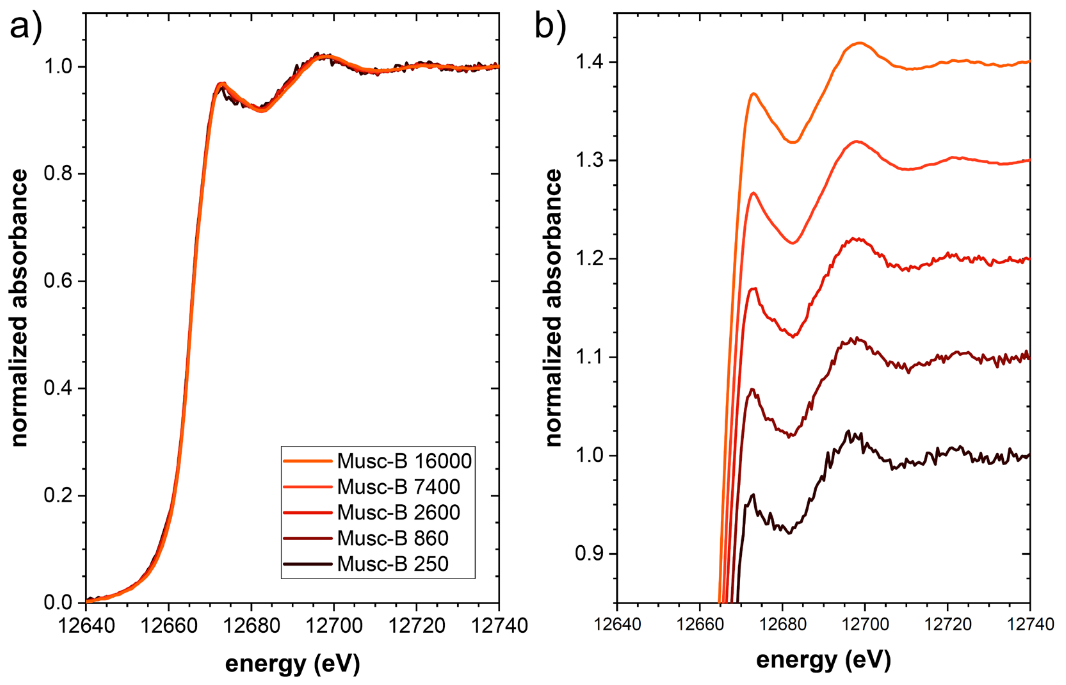

Fig. S10. XANES spectra of Tl adsorbed onto Musc-B (numbers indicate Tl loading in mg/kg). (a) Overlay plot. (b) Zoom into stacked plot with y-axis offset of 0.1 between spectra.

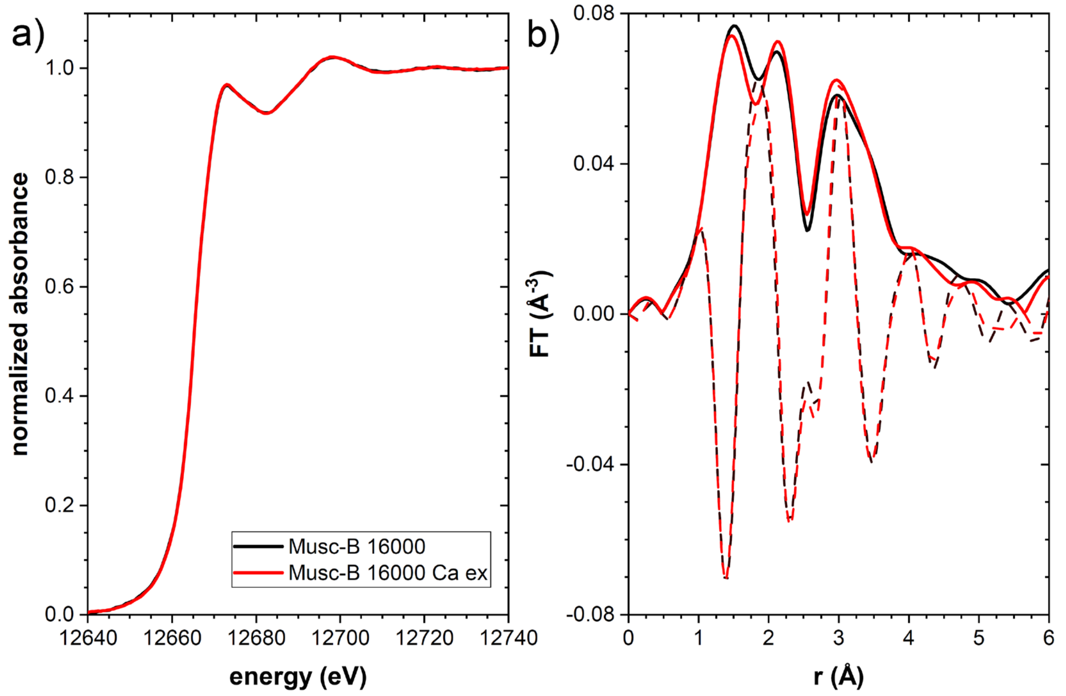

Fig. S11. (a) XANES spectra of Musc-B with 16000 mg/kg adsorbed Tl and analogous sample after extraction of 19% of Tl with 0.1 M $CaCl_2$ (Musc-B 16000 Ca ex). (b) Corresponding Fourier-transformed k^2-weighted EXAFS spectra (solid lines: magnitude; dashed lines: imaginary part).

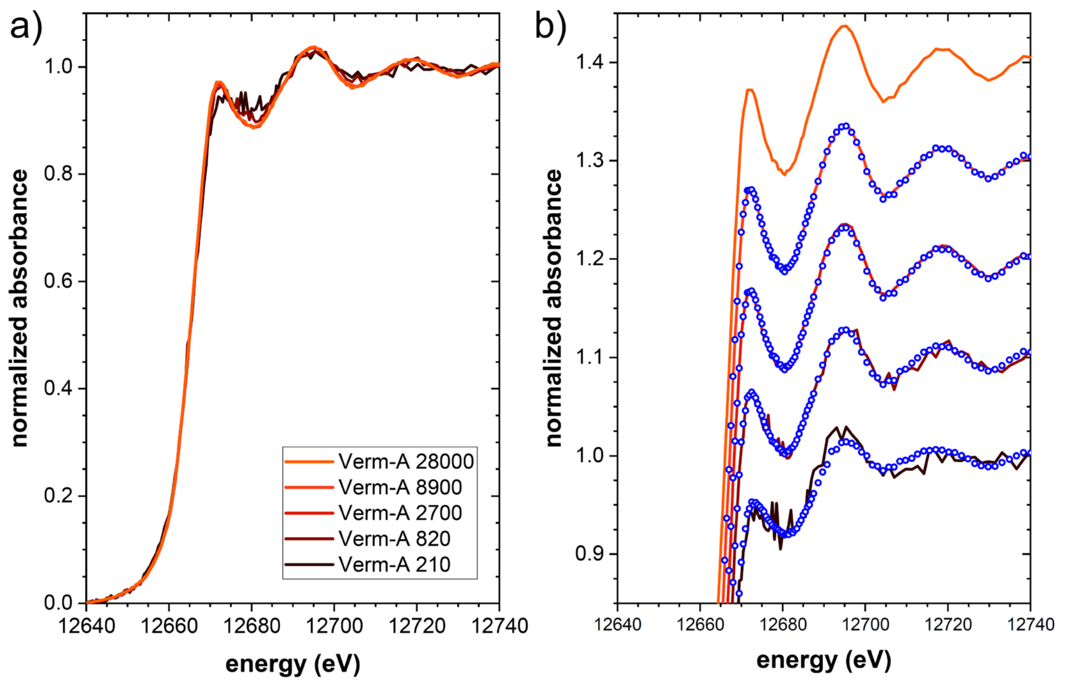

Fig. S12. XANES spectra of Tl adsorbed onto Verm-A (numbers indicate Tl loading in mg/kg). (a) Overlay plot. (b) Zoom into stacked plot with y-axis offset of 0.1 between spectra. Blue-dotted lines represent LCF reconstructions based on LCF results reported in Table S5.

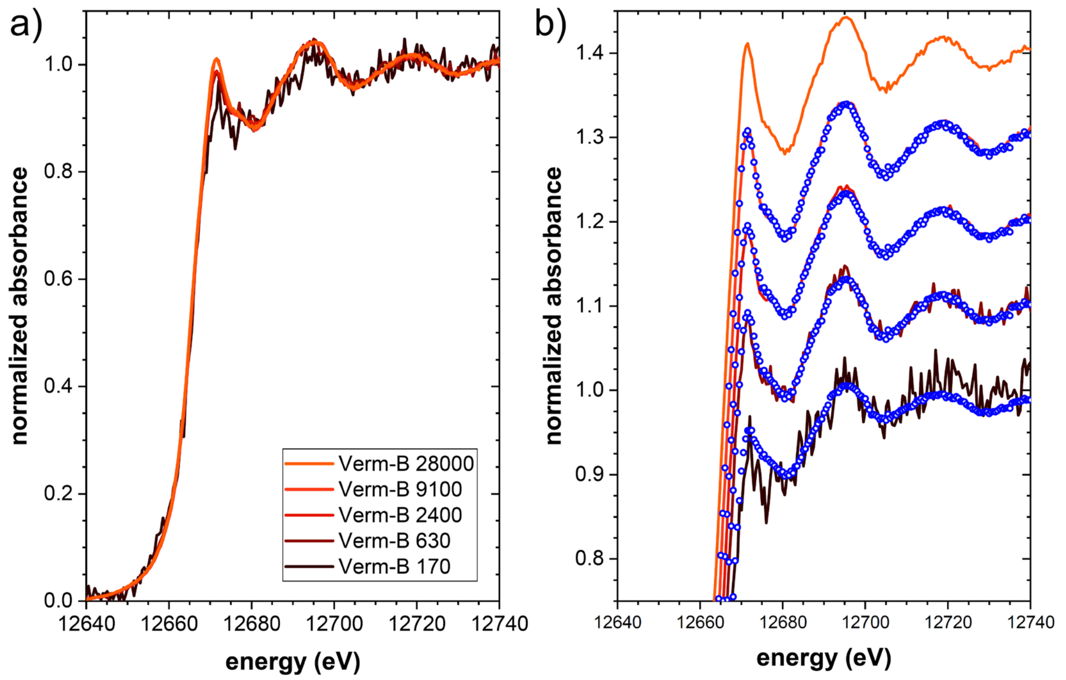

Fig. S13. XANES spectra of Tl adsorbed onto Verm-B (numbers indicate Tl loading in mg/kg). (a) Overlay plot. (b) Zoom into stacked plot with y-axis offset of 0.1 between spectra. Blue-dotted lines represent LCF reconstructions based on LCF results reported in Table S5.

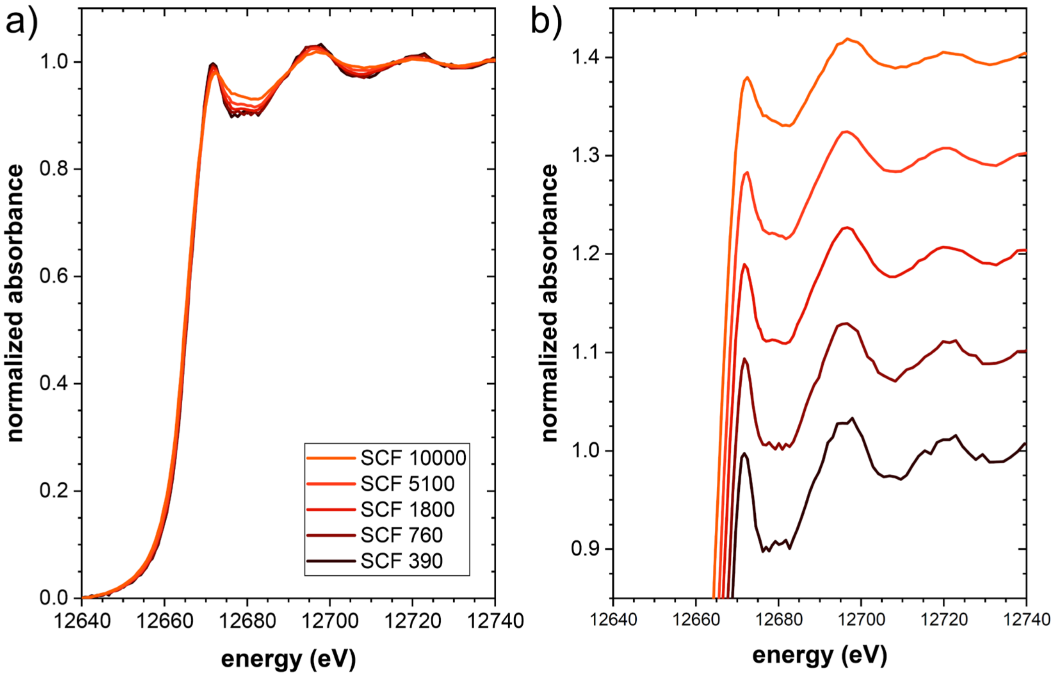

Fig. S14. XANES spectra of Tl adsorbed onto SCF (numbers indicate Tl loading in mg/kg). (a) Overlay plot. (b) Zoom into stacked plot with y-axis offset of 0.1 between spectra.

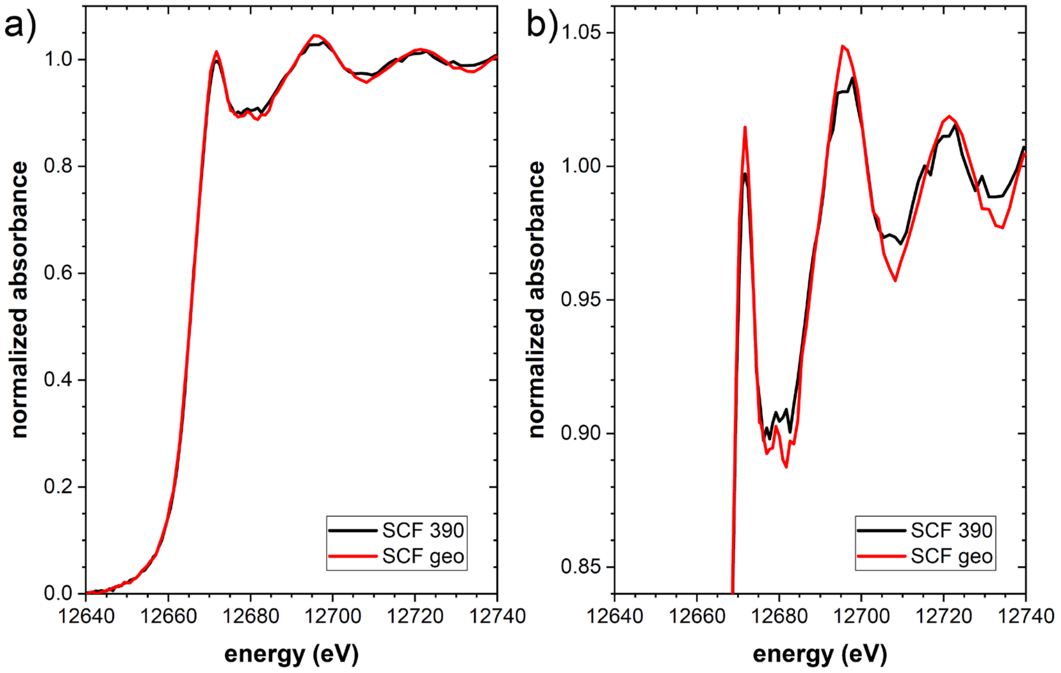

Fig. S15. (a,b) XANES spectra of geogenic Tl in SCF (SCF geo) and of SCF with lowest adsorbed Tl loading (SCF 390). (b) Zoom into XANES post edge oscillations.

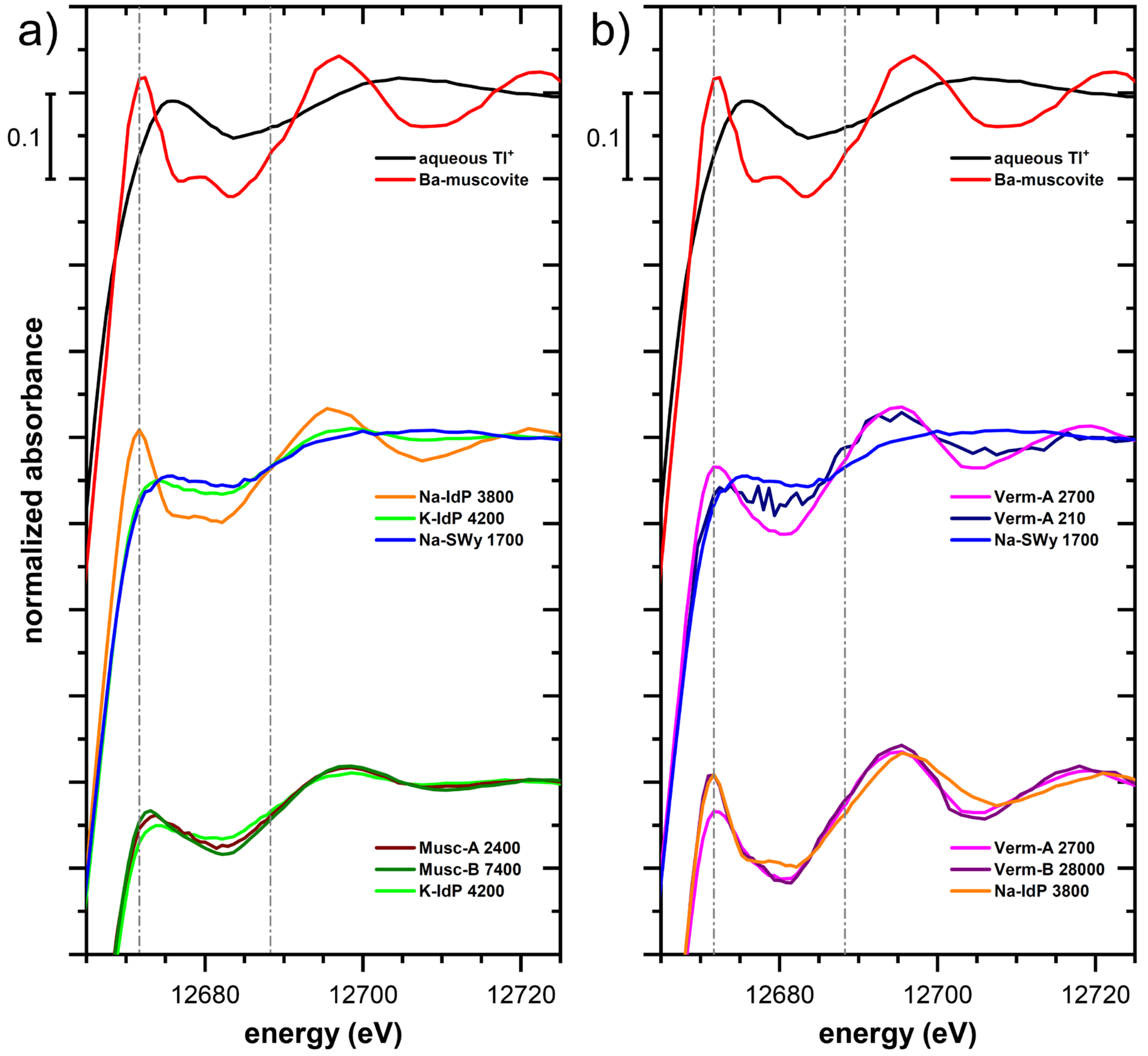

Fig. S16. Comparison of selected XANES spectra in analogy to Fig. 5. Zoom into post-edge region.

3.4. LCF analysis of XANES spectra of Tl-loaded vermiculites

The XANES spectra of the Tl-loaded vermiculites Verm-A and Verm-B showed a spectral shift with increasing Tl loading (Figs. S12 and S13) that was evaluated by LCF. As reference spectra for LCF analysis, the spectrum Na-SWy 1700 and the spectrum of the highest loaded vermiculite sample of the respective series (Verm-A 28000 or Verm-B 28000) were used. The LCF results are listed in Table S5, the LCF reconstructions are shown in Figs. S12 and S13. The results show that the spectral trends can be adequately described by a decreasing fraction of the reference spectrum Na-SWy 1700 with increasing Tl loading, which we interpret as a decrease in the fraction of hydrated Tl^{+} adsorbed in the expanded vermiculite interlayer and a shift towards dominant uptake of dehydrated Tl^{+} in collapsed zones of the vermiculite interlayer.

Table S5. Linear combination fit analysis of the XANES spectra of the Tl-loaded vermiculites Verm-A and Verm-B. The fits were carried out over the energy range 12646-12726 eV. Fitted fractions were constrained to values between 0-1, the sum of the fractions was not constrained. NSSR = normalized sum of squared residuals.

**Sample**	**Na-SWy 1700**	**Verm-A 28000**	**Sum**	**NSSR**
Verm-A 210	0.57	0.44	1.00	0.00071
Verm-A 820	0.25	0.75	1.00	0.00020
Verm-A 2700	0.07	0.93	1.00	0.00007
Verm-A 8900	0.03	0.97	1.00	0.00003
**Sample**	**Na-SWy 1700**	**Verm-B 28000**	**Sum**	**NSSR**
Verm-B 170	0.48	0.51	0.99	0.00367
Verm-B 630	0.19	0.81	1.00	0.00052
Verm-B 2400	0.15	0.85	1.00	0.00029
Verm-B 9100	0.02	0.98	1.00	0.00022

3.5. Shell-fit analysis of selected EXAFS spectra

3.5.1. Ba-muscovite

EXAFS spectra of the Tl-rich Ba-muscovite sample were recorded at room temperature (RT) and 20 K. The k^2-weighted spectra and the corresponding Fourier-transforms (k-range 3-7 $Å^{-1}$; Kaiser-Bessel windows with dk = 2 $Å^{-1}$) together with shell-fits of the peak around 3 Å are shown in Fig. S17. For shell-fit analysis, theoretical scattering paths were calculated using Feff 6.0 [11] as implemented within the Demeter software package [12], based on the structure of illite [13], with structural K replaced by Tl.

The absence of a well-resolved first-shell Tl-O peak can be attributed to the wide distribution of Tl-O distances (as inferred from the illite model) and inherent first-shell disorder in Tl(I)-compounds that can be induced by the lone electron pair of the Tl^+ cation (see also Fig. S5 and related text). The wide spread in first-shell Tl-O distances results in destructive interferences between the backscattering contributions from different O atoms and consequently a strongly attenuated first-shell Tl-O signal. Attempts to fit the weak first-shell peak with a single Tl-O path were unsuccessful, and fits with multiple Tl-O paths could not be constrained as the limited data range strongly limited the number of parameters that could be refined.

We therefore used shell fitting only to test if the marked second-shell peak in the Fourier-transformed spectra of Ba-muscovite could be fitted with a second-shell Tl-Si path. In the fit, the coordination number of second-shell Si was fixed to 12, the expected value for Tl in the interlayer of muscovite, and the amplitude reduction factor to 1. The same interatomic distance and energy shift was fitted for both spectra, whereas separate Debye Waller factors were fitted to account for variations in thermal disorder with temperature. The fit results are listed in Table S6. The fitted Tl-Si distance of 3.84 Å was reasonable, considering an average K-Si distance of 3.77 in illite [13] and that the ionic radius for 12-fold coordinated Tl^+ (1.70 Å) is 0.06 Å larger than for K^+ (1.64 Å) (ionic radii from [14]). Accordingly, we attributed the marked second-shell peak in the EXAFS spectra of Tl in Ba-muscovite to Tl-Si contributions and considered it as diagnostic spectral feature of Tl bound in the siloxane cavities of micaceous clay minerals.

3.5.2. Tl adsorbed onto Na-IdP

In Figure S18, the XANES and EXAFS spectra of samples Na-IdP with 50, 300 and 3800 mg/kg adsorbed Tl are compared (samples from ref[4]). No marked changes are observed among the three XANES spectra, and also the EXAFS spectra of the Na-IdP with 300 and 3800 mg/kg adsorbed Tl do not reveal marked differences. The EXAFS spectrum Na-IdP 50 is very noisy, but

as far as this can be judged, does not seem to vary from the spectra of higher loaded samples. In the 3-site cation exchange model parameterized for Tl adsorption onto Na-IdP, the FES concentration has been set to 0.65 mmol/kg.[4] The sample Na-IdP 50 has a Tl loading of 0.25 mmol/kg Tl, significantly less than the FES concentration in the model, and the sample Na-IdP a Tl loading of 1.5 mmol/kg, 2.3 times the FES concentration. The absence of marked spectral changes from the sample Na-IdP 50 over the sample Na-IdP 300 to the sample Na-IdP 3800 with a much higher Tl loading of 19 mmol/kg suggested that up to 19 mmol/kg Tl could be adsorbed with similar local Tl coordination as low levels of Tl whose adsorption was attributed to high-affinity FES in the 3-site cation exchange model.

The EXAFS spectra of Tl adsorbed onto Na-IdP at loadings of 3800 mg/kg (recorded at room temperature; sample from ref[4]) and 3200 mg/kg (spectrum recorded at 20 K) are shown in Fig. S19. The spectra exhibit a second-shell peak at the same position as Tl-rich Ba-muscovite (Fig. S17). Assuming that Tl adsorbed onto the Na-IdP by complexation between two siloxane cavities, a Tl-Si coordination number of 12 would be expected as for Tl in Ba-muscovite. The much lower intensity of the second-shell peak of the Na-IdP spectra than the Ba-muscovite spectra might thus point to higher structural disorder of freshly adsorbed than structurally incorporated Tl. A shell-fit analysis in analogy to the one for Tl in Ba-muscovite was thus performed to test if the lower peak amplitudes could be attributed to higher disorder. The fit parameters are listed in Table S6, the fits are shown in Fig. S19. The spectra can be reasonably reproduced with a fit based on a Tl-Si coordination number of 12. The much higher Debye Waller parameters than for Tl in Ba-muscovite agree with the postulated higher disorder. Remarkably, the Debye Waller factors of the spectrum recorded at RT (0.038 $Å^2$) and at 20 K (0.025 $Å^2$) encompass the Debye Waller factor for second-shell Si derived from an EXAFS spectrum of Cs bound in the interlayer of illite that had been recorded at 80 K (0.032 $Å^2$),[15] further supporting the fit approach. A longer Tl-Si distance was fit for Tl adsorbed onto illite (3.95 Å) than for Tl in Ba-muscovite (3.84 Å), possibly because the collapsed siloxane cavities at the frayed edges of illite platelets feature a larger spacing than the cavities in the interlayer of muscovite platelets where smaller K^+ is the dominant interlayer cation. Due to the limited fit range, however, the fitted parameters were relatively poorly constrained. Furthermore, the fitted peak positions exhibit slight displacements from the experimental peak position, possibly pointing to spectral contributions from other scattering paths.

3.5.3. *Tl adsorbed onto K-IdP, Na-SWy, Musc-A and Musc-B and Verm-A and Verm-B*

In Fig. S20, the EXAFS spectra of Tl adsorbed on to K-IdP (4200), Na-SWy (1700), Musc-A (2400), Musc-B (7400), Verm-A (2700) and Verm-B (28000) (with loadings in mg/kg) are shown together with shell-fits based on a single Tl-Si path to reproduce the experimental second-shell peak. The fit results are listed in Table S6. These shell-fits served to test if the experimental data could be reasonably reproduced by model fits in which the Tl-Si coordination numbers were set to the ones inferred from the interpretation of the combined macroscopic (adsorption data) and spectroscopic results. Owing to the limited EXAFS data range and the correlation between Tl-Si coordination number and Debye Waller factor, the fits were poorly constrained and fit results need to be interpreted with caution. In the manuscript, the EXAFS spectra shown in Fig. S20 are shown in Figs. 6 and 7, where they are qualitatively discussed with respect to the intensity of the second-shell Tl-Si peak and the position of the first-shell Tl-O peak.

For the K-saturated illite K-IdP and the muscovites Musc-A and Musc-B, the macroscopic and spectroscopic results suggested that Tl was mainly adsorbed by complexation onto trigonal cavities on the planar surfaces. The model fits with a fixed Tl-Si coordination number of 6 (Table S6, fits 3 and 5) reasonably reproduced the experimental data (Fig. S20). The Tl-Si distances fell into the range of those of Ba-muscovite and Na-IdP, and the Debye Waller factors were similar to those of Tl adsorbed onto Na-IdP 3800.

In the case of Tl adsorbed onto Na-SWy, the Tl-Si coordination number was refined and the Debye Waller parameter was arbitrarily set to the one obtained for the spectrum Na-IdP 3800. The modelled Tl-Si peak closely reproduced the experimental peak.

For Verm-A 2700 and Verm-B 28000, the macroscopic and spectroscopic data suggested that Tl was mainly adsorbed as dehydrated Tl^{+} complexed between two siloxane cavities in the collapsed interlayer. Accordingly, the CN of the Tl-Si path was set to 12 in the model fits, as for Na-IdP (Table S6). Especially for Verm-B 28000, the difference between modelled and experimental peak shape and amplitude was larger than for Na-IdP 3800 (Fig. S19, S20). Using a single Tl-Si path, the discrepancies could not be further reduced, as a higher Debye Waller factor would have been required to broaden the modelled peaks, but a lower one to obtain a higher peak amplitude. This points to additional spectral contributions, potentially arising from two distinct Tl-Si shells, from second-nearest O atoms, or from multiple scattering paths. However, the limited EXAFS data range did not allow to further test these possibilities because the number of adjustable fit parameters could not be further increased.

Table S6. Shell-fit analysis of selected EXAFS spectra using a Tl-Si backscattering path (CN = coordination number; δ^2 = Debye Waller factor, R = interatomic distance, dE_0 = energy shift, r-factor = normalized sum of squared residuals of fit). Spectra with same Fit. Nr. (and same background color) were fit simultaneously with some parameters constrained to be the same for both spectra. Values given without fit uncertainty (in parentheses) were fixed. The amplitude reduction factor was set to 1 in all fits. Fits were conducted on the Fourier-transformed k^2-weighted EXAFS spectra (k-range 3-7 $Å^{-1}$; Kaiser Bessel window parameter 2 $Å^{-1}$) over the r-range 2.5-3.8 Å. The number of independent datapoints was 6.3 in fits including two spectra, and 3.2 in fits of one spectrum. The sample spectra and fits are shown in Figs. S17, S19 and S20.

Fit 1: Spectra of Ba-muscovite recorded at room temperature (RT) and 20 K. CN set to 12 assuming Tl to substitute K between two siloxane cavities. Individual δ^2 (temperature-dependence), but same R for both spectra.

Fit 2: Spectra of 3800 / 3200 mg/kg Tl adsorbed onto Na-IdP recorded at RT and 20 K. CN set to 12 assuming Tl to be complexed between two siloxane cavities. Individual δ^2 (temperature-dependence), but same R for both spectra.

Fit 3: Spectrum of Tl adsorbed onto K-IdP. CN set to 6 assuming Tl to be complexed on siloxane cavity. dE_0 fixed at value from Fit 2.

Fit 4: Spectrum of Tl adsorbed onto Na-SWy. CN refined, δ^2 and dE_0 set to values from Fit 2.

Fit 5: Spectra of Musc-A with 2400 and Musc-B with 7400 mg/kg adsorbed Tl. CN set to 6 assuming Tl to be complexed on siloxane cavity. Same δ^2 for both spectra, but individual R. dE_0 set to value of Fit 2.

Fit 6: Spectra of Verm-A with 2700 and Verm-B with 28000 mg/kg adsorbed Tl. CN set to 12 assuming Tl to be complexed between two siloxane cavities. Same δ^2 for both spectra, but individual R. dE_0 set to value of Fit 2.

**Spectrum**	**Fit Nr.**	**CN**	**δ^2**	**R**	**dE_0**	**r-factor**
			($Å^2$)	(Å)	(eV)	
Ba-muscovite (RT)	1	12[a]	0.016 (0.006)	3.84 (0.05)	5 (3)	0.082
Ba-muscovite (20 K)	1		0.007 (0.004)			
Na-IdP 3800 (RT)	2	12[a]	0.038 (0.015)	3.95 (0.07)	8 (3)	0.096
Na-IdP 3200 (20 K)	2		0.025 (0.005)			
K-IdP 4200 (RT)	3	6	0.043 (0.006)	3.96 (0.03)	8	0.069
Na-SWy 1700 (RT)	4	3.6 (1.4)	0.038	4.04 (0.04)	8	0.154
Musc-A 2400 (RT)	5	6	0.039 (0.005)	3.91 (0.06)	8	0.105
Musc-B 7400 (RT)	5			3.86 (0.02)		
Verm-A 2700 (RT)	6	12[a]	0.031 (0.006)	3.99 (0.03)	8	0.166
Verm-B 28000 (RT)	6			4.00 (0.05)		

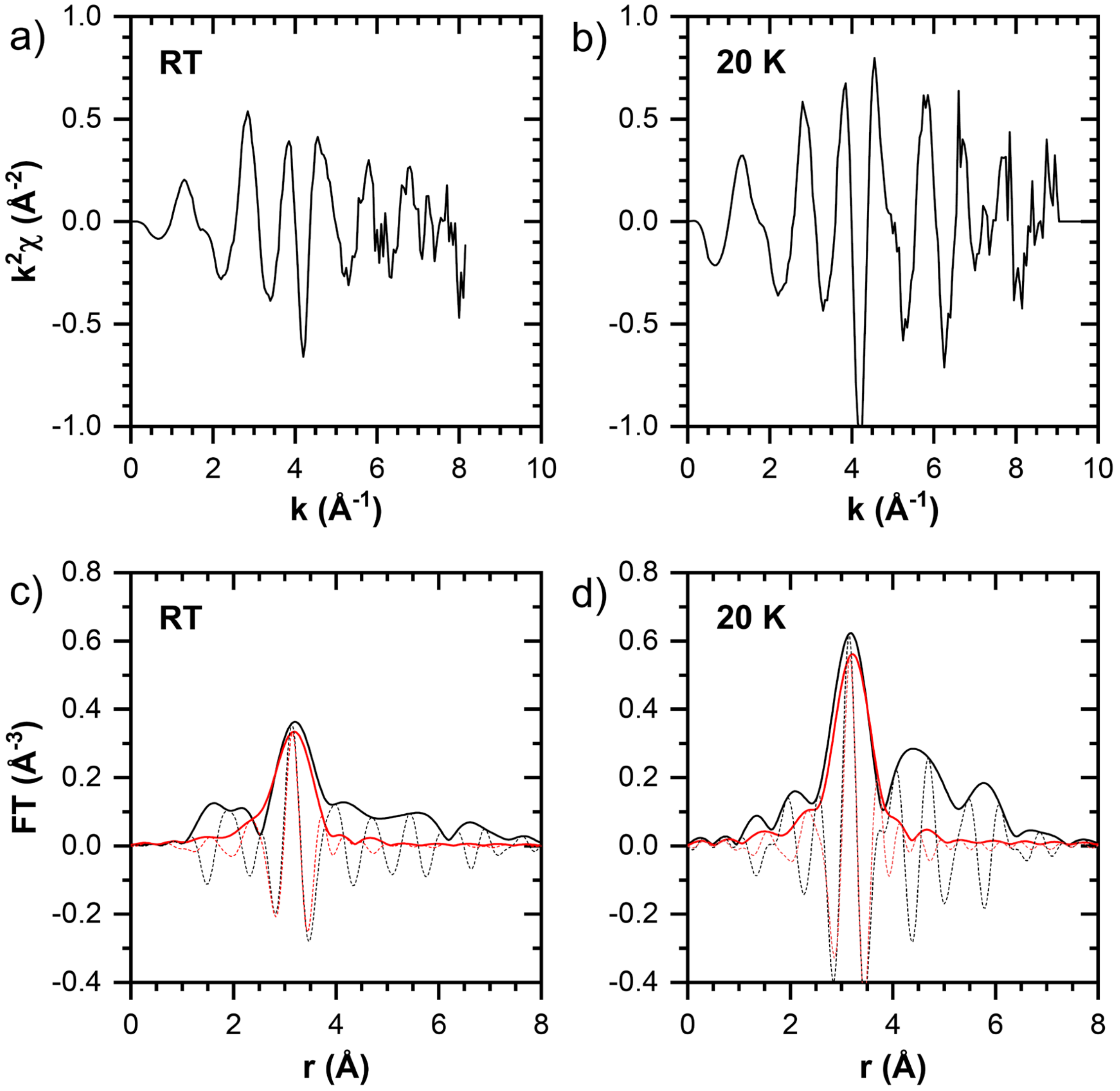

Fig. S17. (a, b) k^2-weighted EXAFS spectra of Tl-rich Ba-muscovite recorded at room temperature (RT) and 20 K and (c, d) the corresponding Fourier-transforms (k-range 3-7 Å, Kaiser-Bessel window with dk = 2.0 Å). The red lines in (c,d) represent the fitted contribution of 12 second-shell Si atoms at 3.84 Å from Tl (parameters in Table S6).

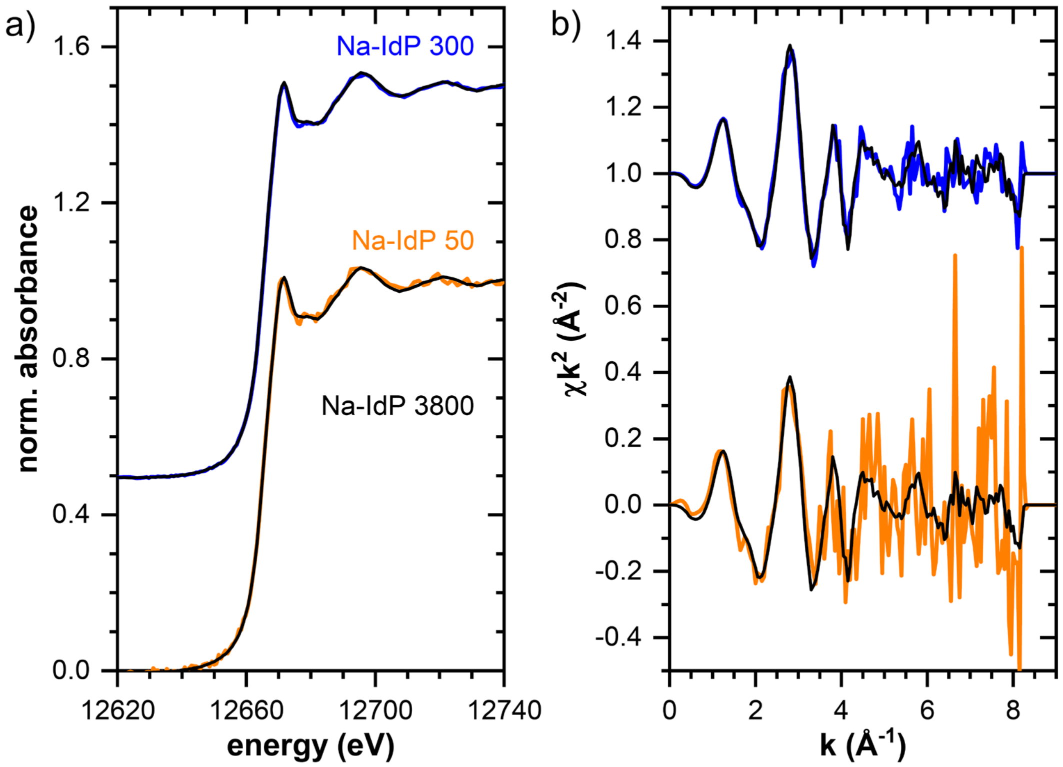

Fig. S18. Comparison of the (a) normalized XANES spectra and (b) k^2-weighted EXAFS spectra of the samples Na-IdP 300 (1.5 mmol/kg Tl) and Na-IdP 50 (0.25 mmol/kg Tl) with the spectrum Na-IdP 3800 (19 mmol/kg Tl). The XANES and EXAFS spectra of the sample Na-IdP 300 are offset by +0.5 and +1.0, respectively.

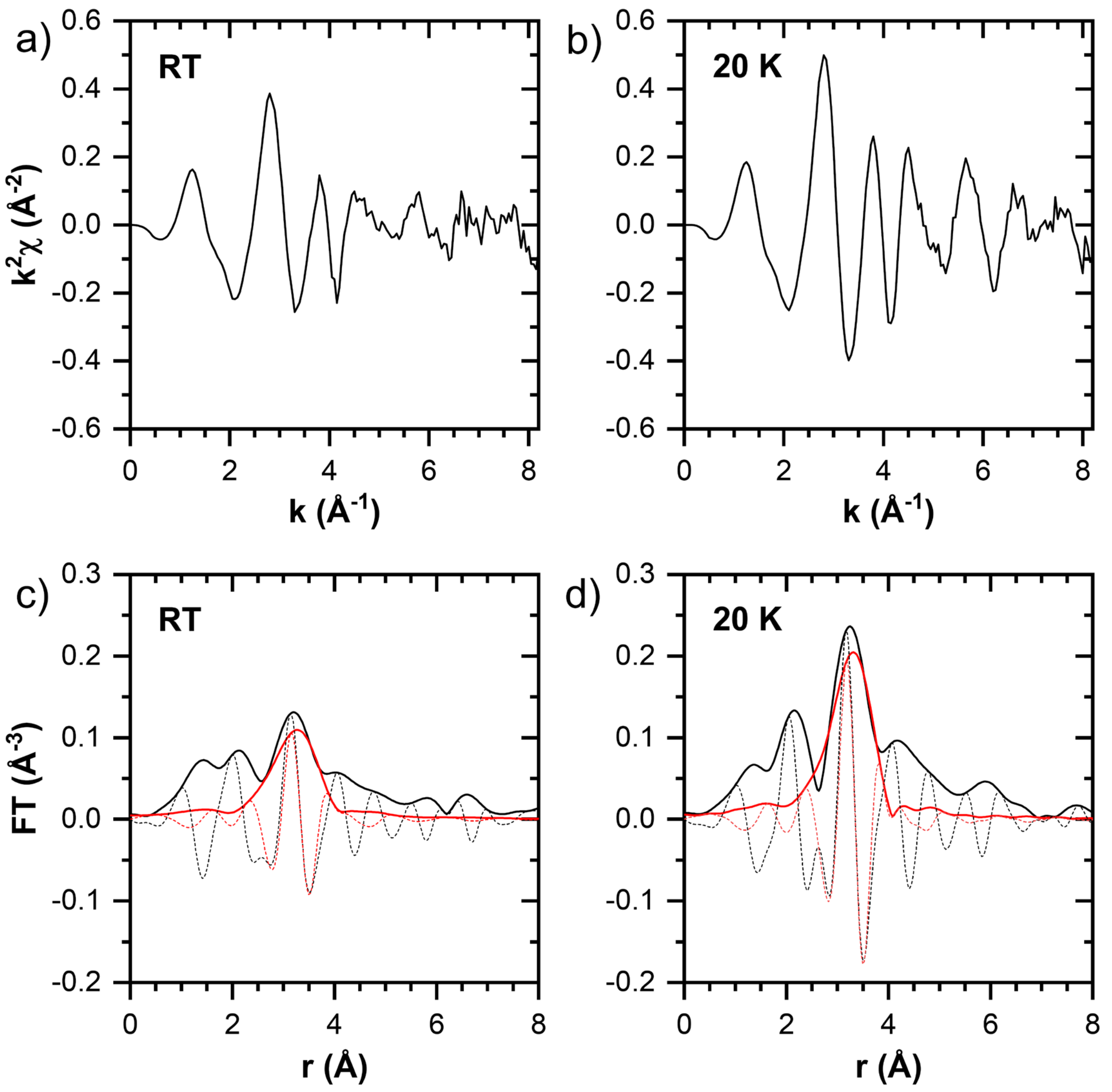

Fig. S19. (a, b) k^2-weighted EXAFS spectra of Tl(I) adsorbed onto Na-IdP recorded at RT (3800 mg/kg Tl; sample from Wick et al.(2018)[4]) and 20 K (3200 mg/kg; sample Na-IdP 3200 from this study), and (c, d) the corresponding Fourier-transforms (k-range 3-7 Å, Kaiser-Bessel window with dk = 2.0 Å). The red lines in (c,d) represent the fitted contribution of 12 second-shell Si at 3.95 Å (parameters in Table S6).

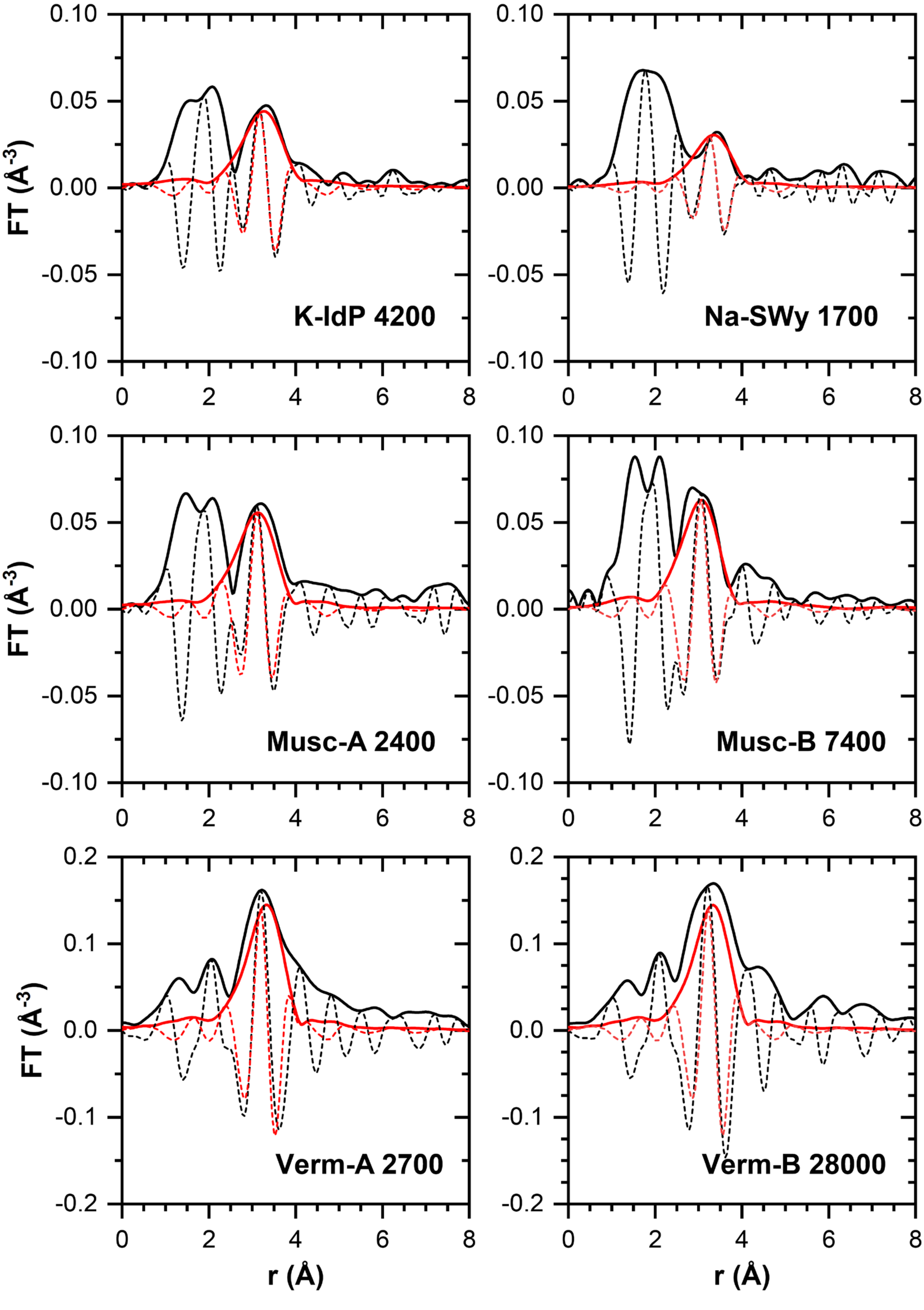

Fig. S20. Fourier-transformed k^2-weighted EXAFS spectra of Tl(I) adsorbed onto K-IdP (4200 mg/kg), Na-SWy (1700 mg/kg), Musc-A (2400 mg/kg), Musc-B (7400 mg/kg), Verm-A (2700 mg/kg) and Verm-B (28000 mg/kg) (black solid lines: Fourier-transform magnitude, black dashed lines: imaginary parts). Red lines represent shell-fits of the second-shell peak using a Tl-Si path, with the fit parameters listed in Table S6.

4. References

1. A. Steudel, P. G. Weidler, R. Schuhmann and K. Emmerich, Cation exchange reactions of vermiculite with Cu-triethylenetetramine as affected by mechanical and chemical pretreatment, *Clays Clay Min.*, 2009, **57**, 486-493.
2. A. Voegelin, N. Pfenninger, J. Petrikis, J. Majzlan, M. Plötze, A.-C. Senn, S. Mangold, R. Steininger and J. Göttlicher, Thallium speciation and extractability in a thallium- and arsenic-rich soil developed from mineralized carbonate rock, *Environ. Sci. Technol.*, 2015, **49**, 5390-5398.
3. B. Baeyens and M. H. Bradbury, Cation exchange capacity measurements on illite using the sodium and cesium isotope dilution technique: Effects of the index cation, electrolyte concentration and competition: Modeling, *Clays Clay Min.*, 2004, **52**, 421-431.
4. S. Wick, B. Baeyens, M. Marques Fernandes and A. Voegelin, Thallium adsorption onto illite, *Environmental Science Technology*, 2018, **52**, 571-580.
5. B. Baeyens and M. H. Bradbury, A mechanistic description of Ni and Zn sorption on Na-montmorillonite, Part I:Titration and sorption measurements, *J. Contam. Hydrol.*, 1997, **27**, 199-222.
6. L. A. Martin, A. Wissocq, M. F. Benedetti and C. Latrille, Thallium (Tl) sorption onto illite and smectite: Implications for Tl mobility in the environment, *Geochim. Cosmochim. Acta*, 2018, **230**, 1-16.
7. D. L. Sparks, *Environmental Soil Chemistry*, Academic Press, San Diego, 2003.
8. D. L. Dolcater, M. L. Jackson and J. K. Syers, Cation exchange selectivity in mica and vermiculite, *Am. Min.*, 1972, **57**, 1823-1831.
9. R. C. Mackenzie, A micromethod for the determination of cation-exchange capacity of clay, *Journal of Colloid Science*, 1951, **6**, 219-222.
10. S. Wick, B. Baeyens, M. Marques Fernandes, J. Göttlicher, M. Fischer, N. Pfenninger, M. Plötze and A. Voegelin, Thallium sorption and speciation in soils: Role of micaceous clay minerals and manganese oxides, *Geochim. Cosmochim. Acta*, 2020, **288**, 83-100.
11. J. J. Rehr, R. C. Albers and S. I. Zabinsky, High-order multiple-scattering calculations of X-ray absorption fine structure, *Physical Review Letters*, 1992, **69**, 3397-3400.
12. B. Ravel and M. Newville, ATHENA, ARTEMIS, HEPHAESTUS: data analysis for X-ray absorption spectroscopy using IFEFFIT, *J. Synchrotron Rad.*, 2005, **12**, 537-541.
13. V. A. Drits, B. B. Zviagina, D. K. McCarthy and A. L. Salyn, Factors responsible for crystal-chemical variations in the solid solutions from illite to aluminoceladonite and from glauconite to celadonite, *Am. Min.*, 2010, **95**, 348-361.
14. R. D. Shannon, Revised effective ionic radii and systematic studies of interatomic distances in halides and chalcogenides, *Acta Crystallographica A*, 1976, **32**, 751-767.
15. A. J. Fuller, S. Shaw, M. B. Ward, S. J. Haigh, J. F. W. Mosselmans, C. L. Peacock, S. Stackhouse, A. J. Dent, D. Trivedi and I. T. Burke, Caesium incorporation and retention in illite interlayers, *Applied Clay Science*, 2015, **108**, 128-134.
